# Rapid Nanocellulose Wet Nanoimprint Lithography for Tunable Structural Color

**DOI:** 10.1021/acsnano.5c15904

**Published:** 2025-12-23

**Authors:** Hui Mao, Zain Ahmad, Lu Xin, Hisay Lama, Pengfei Fan, Philip Shields, Samuel Eyley, Wim Thielemans, João T. Cabral

**Affiliations:** † Department of Chemical Engineering, 4615 Imperial College London, London SW7 2AZ, U.K.; ‡ Centre for Nanoscience and Nanotechnology, 1555 University of Bath, Bath BA2 7AY, U.K.; § Sustainable Materials Lab, Department of Chemical Engineering, 97541 KU Leuven Kulak Kortrijk Campus, E. Sabbelaan 53, Kortrijk 8500, Belgium

**Keywords:** nanocellulose, cellulose nanocrystal, patterning, nanoimprint lithography, structural color

## Abstract

We report a nanopatterning approach for nanocellulose films capable of generating structural color, within time scales of a few minutes, 3–4 orders of magnitude faster than typical cellulose nanocrystal (CNC) self-assembly into chiral nematic structures. We employ a wet-nanoimprint lithography (wet-NIL) approach, in which a prescribed mold is imprinted onto nanocellulose suspensions, supported by a semipermeable membrane that enables water removal during pattern transfer, and thus film formation. We examine the roles of nanocellulose type, concentration, rheology, imprint pressure, and temperature, as well as pattern geometry, seeking to reduce wet-NIL time scales while ensuring replication fidelity. The dynamic response and durability of the patterned CNC films are examined by light scattering and microscopy over multiple humidity cycles, direct contact with water droplets, and long-term storage (up to 12 months). We then model and optimize the diffractive color selection and vibrancy of the patterned CNC films, yielding the largest angular range of single colors and providing a sustainable approach for CNC structure color, which is benchmarked against chiral self-assembly and hybrid patterning methods.

## Graphical abstract

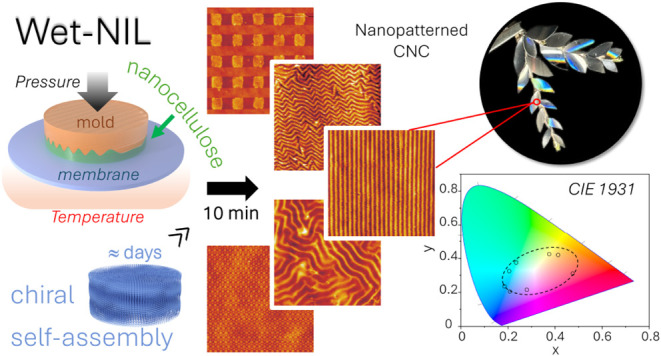

## Introduction

Structural color, generated by interference and diffraction of visible light by periodic nanostructures, is ubiquitous in nature, providing vivid, nonfading coloration (by contrast with chemical pigmentation), with multifaceted function.^1–3^ In Hibiscus trionum, for instance, directional iridescence from floral striations enhances visual cues for pollinators, improving flower detectability, orientation, and species recognition.^4–7^ Structural color can arise from various optical mechanisms, including thin-film interference, multilayer reflection, bulk photonic crystal diffraction as well as surface diffraction,^8^ which have been technologically exploited in environmental sensing, anticounterfeiting, and decorative materials.^9,10^

Cellulose is the most abundant (bio)­polymer on Earth.^11,12^ Among cellulose-derived materials, nanocellulose (NC) is a class of nanomaterials exhibiting at least one dimension in the nanoscale; these include cellulose nanocrystals (CNCs), cellulose nanofibrils (CNFs), and bacterial nanocellulose (BNC) that can be obtained from the lignocellulosic biomass (including from forest and agricultural residues) or produced by bacteria. NC materials comprise cellulose chains bundled together into semicrystalline bundles, isolated as nanorods or nanofibrils. In addition to their intrinsically abundant and sustainable nature, NC exhibits high aspect ratio, exceptional mechanical and thermal properties, chemical inertness and suitability for chemical functionalization, as well as biodegradability and nontoxicity.^13–15^ NC has thus emerged as a sustainable alternative to petroleum-based polymers and resource-insensitive inorganic materials, in applications ranging from packaging and textiles to advanced optics and electronics.^11^

CNCs dispersed in water behave as rod-like colloids whose liquid crystalline nature can result in the formation of chiral nematic solid films via cholesteric self-assembly.^16–18^ Such helicoidal periodic stacks selectively reflect circularly polarized light, producing iridescent structural colors that depend on viewing angle and the pitch of the structure.^19–22^ The optical properties of CNC self-assembled films, generally formed by slow solvent evaporation and kinetic arrest, can be tailored by controlling factors such as CNC concentration, ionic strength, additives (typically polymers and solvents), and the application of external fields.^23–25^ This yields films with tunable wavelength reflection, polarization control, and environmental responsiveness.^26^ Despite the intrinsic optical properties of CNCs, widespread deployment remains limited by slow self-assembly kinetics of chiral nematic films (days to weeks), poor water resistance, and limited structural control.^27–29^ A rapid, robust, and scalable fabrication approach is needed to unlock the potential of CNC as a sustainable alternative to traditional colorants.^30–33^ Recent developments have included the generation of CNC ’pigments’ via the processing of ordered microparticles and fragmented films.^34^

Direct nanoimprint lithography (direct-NIL) is a mature, scalable, and low-cost fabrication technique to achieve diffraction-based color.^35^ However, its application to CNC patterning has been constrained by the high water content in CNC suspensions (and thus lengthy film formation time scales), or conversely by the shape recovery of imprinted dried NC films, associated with the dry film’s elasticity and compromising pattern fidelity.^36,37^ The cholesteric self-assembly can also be achieved atop a grating surface (or mold, sometimes referred to as “inverse”-NIL) to achieve additional optical functionalities by diffraction-based structural coloration,^38–41^ where periodic surface patterns scatter light at specific angles determined by pattern dimensions, offering material-independent color control.^42–45^ However, drying-induced deformation (which hinders conformal mold contact and thus yields low pattern fidelity) and slow solvent evaporation limit the applicability of this technology to CNC dispersions.

In this work, we introduce a rapid and facile strategy for fabricating structurally colored CNC films via an approach we term ‘wet direct nanoimprint lithography’, or wet-NIL. By incorporating a water-permeable planar support that enables moisture removal, we demonstrate the direct imprinting onto CNC suspensions. Systematically optimizing process parameters (temperature, pressure, concentration, and duration), we achieve high-fidelity surface patterning (92%) with processing times down to 10 min. The resulting nanopatterned CNC films exhibit tunable, angle-dependent structural color governed by the geometry of the imprinted nanostructures. Further, these films demonstrate excellent environmental stability, retaining optical functionality through repeated humidity cycling and direct water exposure. This approach provides a sustainable, scalable route to CNC-based photonic materials, addressing growing demand for bioderived environmental sensing, anticounterfeiting, and coating technologies, offering a compelling alternative to conventional pigment-based coloration.

## Results and Discussion

### CNC Nanopatterning by Wet-Nanoimprint Lithography (wet-NIL)

Cellulose nanocrystals (CNC) with characteristic rod-like morphology (width: 6.3 ± 1.6 nm, length: 176 ± 65 nm) were characterized by transmission electron microscopy (TEM) and dynamic light scattering (DLS), Figures 1a–d and S1, as well as wide-angle X-ray scattering (WAXS), yielding a crystallinity index (CI) of 78% (Figures 1e and S2). The stability of the CNC suspension is predominantly governed by surface charges, with carboxyl groups present at a concentration of 908 μmol/g, as determined via conductometric titration (Figure S3), and a ζ-potential of −50 ± 3 mV (for 0.1 wt % CNC suspension). Cellulose nanofibrils (CNF) and bacterial nanocellulose (BNC), detailed in Figure S4, were also evaluated for imprinting.

**Figure 1:**
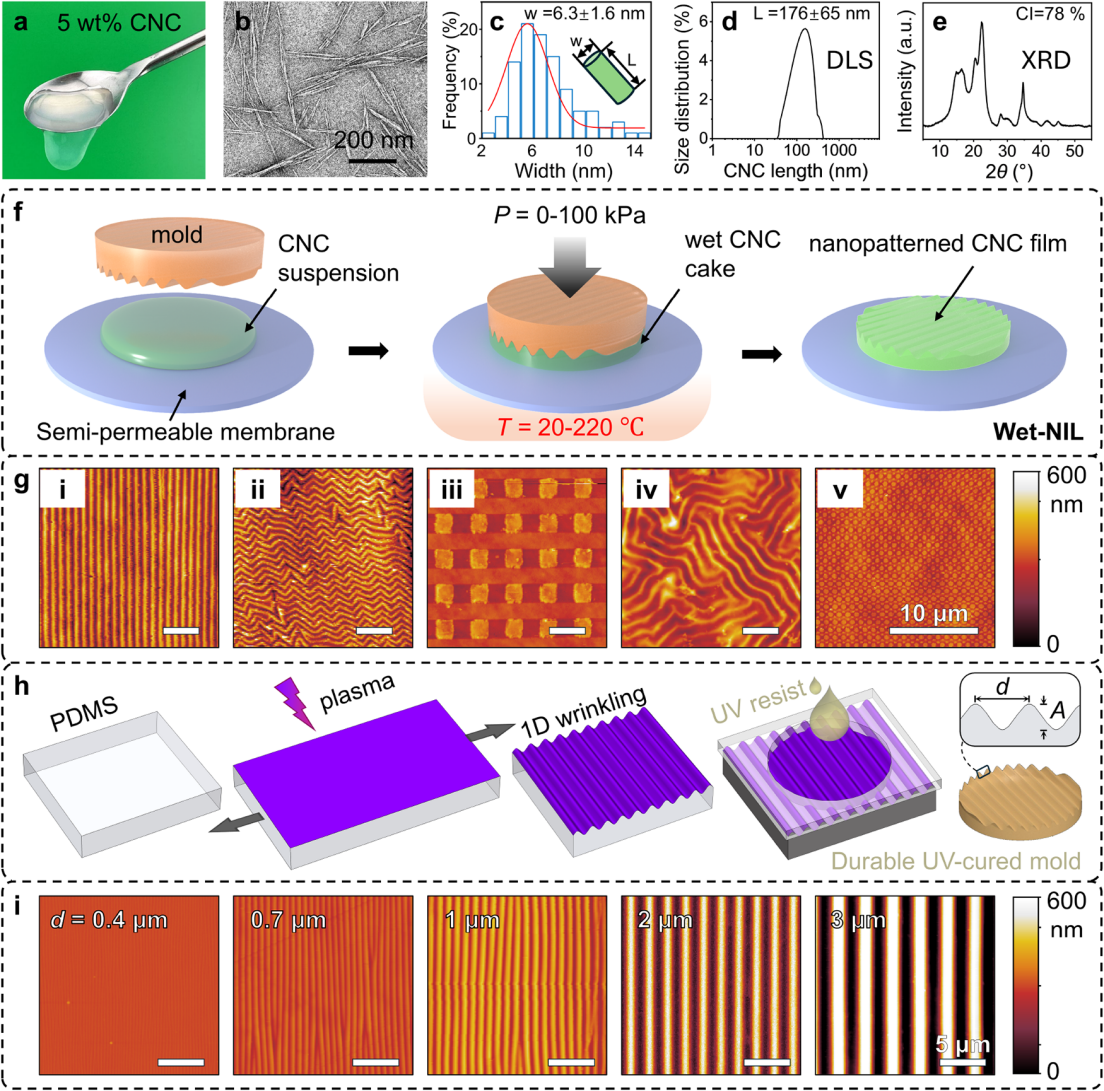
Wet nanoimprinting lithography (Wet-NIL) of CNC suspensions into nanopatterned films. (a) 5 wt % CNC aqueous suspension. (b) TEM micrograph of CNC suspension (provided by Y. Nevo, Melodea). (c) The width distribution of CNC nanorods estimated from TEM, indicating a width of ≈6.3 nm. (d) Population histogram computed by DLS, indicating a length (*L*) ≈ 180 nm. (e) X-ray diffraction pattern of CNC films, with crystallinity index (CI) ≈ 78%. (f) Schematic of the wet-NIL process employing a semipermeable support membrane, and the simultaneous application of pressure *P* at fixed temperature *T*. (g) Atomic force microscopy (AFM) images of the resulting CNC films fabricated by wet-NIL: (i) one-dimensional (1D) lines, (ii) chevron patterns, (iii) square post array, (iv) isotropic pattern, (v) checkerboard. and (h) schematic of fabrication of poly­(dimethylsiloxane) (PDMS) wrinkled patterns, with periodicity *d* and amplitude *A*, and replication onto a photopolymer network. (i) Series of 1D patterns on CNC films fabricated by wet-NIL employing wrinkling templates of varying profiles. A detailed sinusoidal line profile is shown in Figure S10.

The wet-NIL process (illustrated in Figure 1f) involves depositing the CNC suspension between the imprint mold and a planar membrane, semipermeable to water, thus enabling direct moisture evaporation; subsequent application of pressure (ranging between 0 and 100 kPa in this study) and heat (20–220 °C) readily produces nanopatterned films. A range of candidate materials for the support membrane were evaluated in terms of water permeability and transport kinetics, planarity, stability, and ease of removal, and a porous poly­(vinylidene fluoride) (PVDF) mesh was found to provide optimal results (Table S1). The versatility of our approach is illustrated by the diverse nanopatterned architectures achieved, including 1D lines, chevron patterns, square post array, isotropic pattern, and checkerboard, with precise dimensional control and high fidelity (Figure 1g). While imprint molds can be fabricated using a variety of photolithographic and etching methods (Figure S5), we have also evaluated surface wrinkling of oxygen plasma-treated poly­(dimethylsiloxane) (PDMS)^46^ to fabricate a series of molds for wet-NIL. Plasma oxidation generates a bilayer structure consisting of the flexible PDMS substrate topped with a thin glassy skin of thickness *h.* ^47–49^ Subjected to mechanical strain, this bilayer configuration provides the necessary stiffness contrast for controlled wrinkling, whose dimensions are readily tuned by adjusting plasma exposure (which sets *h*) and strain (ϵ). In the high deformation regime,^50^ one-dimensional (1D) surface wrinkling yields sinusoidal patterns with in-plane periodicity (*d*) and amplitude (defined as *A*/2, according to Figure 1h), following

1 $$d=\frac{2\pi h{\left({\mathrm{E̅}}_{\mathrm{skin}}/3{\mathrm{E̅}}_{\mathrm{PDMS}}\right)}^{1/3}}{(1+\epsilon ){\left(1+\xi \right)}^{1/3}}$$

2 $$A/2=\frac{h{\left(\epsilon /\epsilon_{c}-1\right)}^{1/2}}{{\left(1+\epsilon \right)}^{1/2}{\left(1+\xi \right)}^{1/3}}$$

where *E̅*_skin_ and *E̅*_PDMS_ denote the plane strain Young’s moduli of the glassy skin and PDMS substrate (*E̅* = *E*/(1 – ν^2^)), where *E* is the Young’s modulus and ν is the Poisson ratio; ξ = 5ϵ (1 + ϵ) /32, which accounts for the nonlinearity in the stress–strain relationship of the PDMS substrate. A critical strain value ϵ_c_ = (3 *E̅*_PMDS_/*E̅*_skin_)^2/3^/4 must be exceeded to excite the instability, typically ∼ 0.04% in our experiments, with *E*_skin_ ≈ 40 GPa and *E*_PDMS_ ≈ 1.5 MPa, ν ≈ 0.5 for PDMS and 0.2 for the glassy skin.^48^ Pattern dimensions of the mold (Figure S7a) were adjusted by varying strain, plasma power, exposure time, and O_2_ pressure (detailed in Table S2 and Figure S8). To improve mold durability and pattern transfer onto CNC films, the PDMS molds were replicated onto a thiolene-based UV-curable resin (NOA 81, Figure S7b). The overall fabrication process is depicted in Figure 1h, along with a demonstration of varying pattern periodicity in Figure 1i (and Figure S9 and Table S3). We have found CNF and BNC to be comparatively less compatible with wet-NIL (as detailed in Figure S11).

### Wet-NIL Process Optimization

A judicious combination of so-called “flowability” and high replication fidelity is required for wet-NIL of nanopatterned CNC films. As illustrated in Figure 2a, the flowability decreased significantly with increasing CNC concentration from 4 to 12 wt %, which we quantify with rheology measurements (Figure 2b), which exhibit the characteristic shear-thinning behavior of CNC suspensions. Large zero-shear viscosities, corresponding to CNC concentrations above 10 wt %, lead to poor spreading and poor integrity of the resulting films (Figure S12). Further, film drying times, at fixed applied pressure *P* = 10 kPa and temperature *T* = 180 °C, increase with decreasing CNC content (Figure 2c), as expected from the combined effects of higher water content and lower dispersion viscosity. A CNC concentration of 8 wt % was identified as optimal for the wet-NIL process. In addition to concentration, the imprinting temperature largely controls the solvent evaporation kinetics, and processing times were found to exhibit a nonlinear relationship with the temperature, where a minimum of ≈ 10 min was required for complete feature development, after which minimal additional improvements occurred. The temperature–time window (Figure 2d) also identifies an optimal processing temperature of 180 °C (marked by the red star), which balances rapid material processing while preserving material integrity. At this temperature, thermal degradation is avoided, which becomes pronounced at temperatures above 220 °C, as confirmed by the thermogravimetric analysis (TGA) (Figure 2e), thereby establishing the upper threshold for the processing temperature.

**Figure 2:**
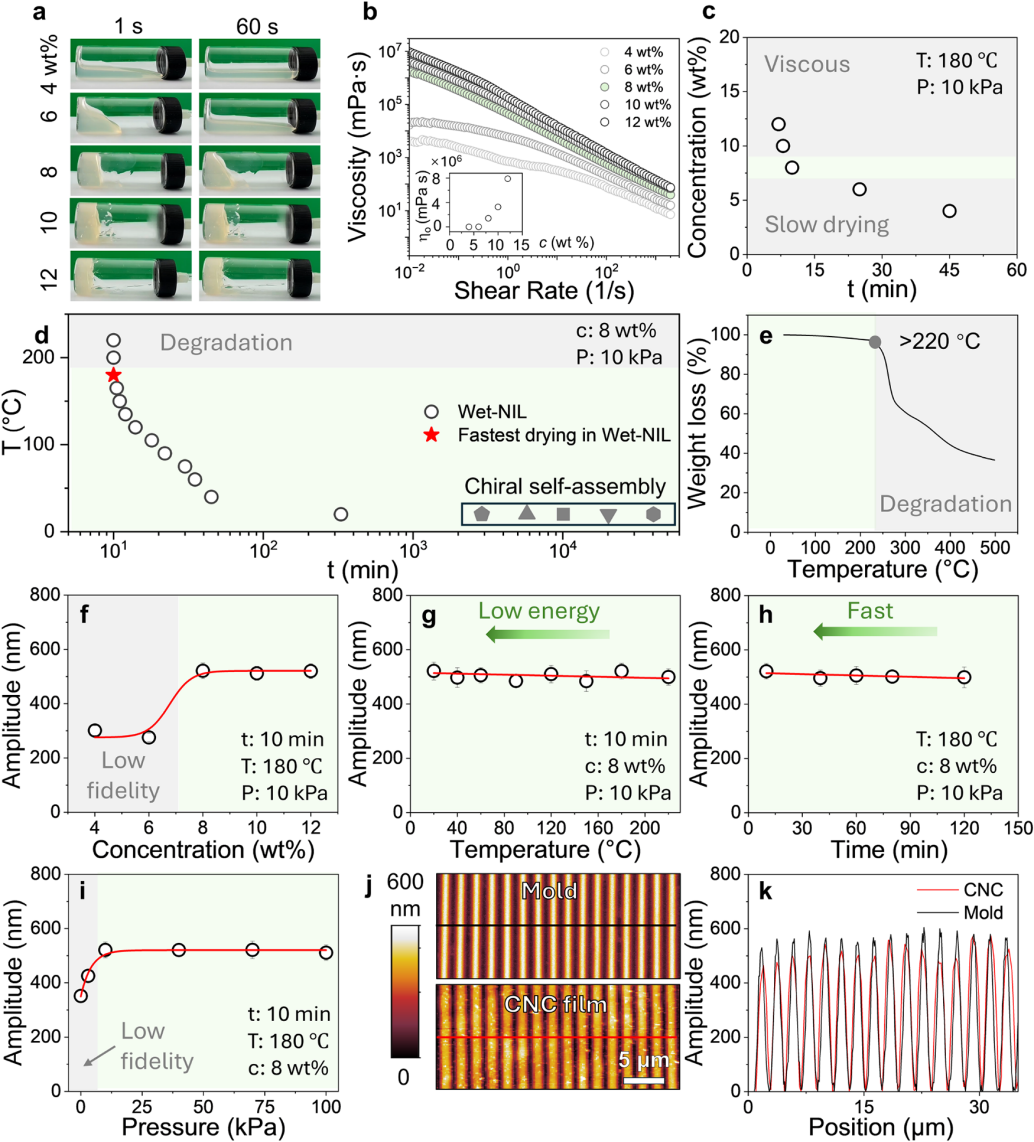
Optimization and process parameters for wet-NIL fabrication of CNC films. (a) Temporal flow behavior of CNC suspensions (4–12 wt %) at 1 and 60 s intervals. (b) Viscosity-shear rate relationships with concentration-dependent rheological profiles; inset shows zero-shear viscosity scaling. (c) Drying time evolution on CNC concentration during thermal drying (180 °C). (d) Drying time to temperature window showing optimal fabrication time at CNC concentration of 8 wt % and pressure of 10 kPa, and comparing with literature data of CNC chiral self-assembly process ⬠,^38^ △,^19^ □,^51^▽,^52^ ⬡.^53^ (e) TGA curve of CNC. (f–i) Pattern amplitude dependence on concentration, temperature, time, and pressure, respectively, defining optimal processing conditions (8 wt %, 180 °C, 10 kPa, 10 min). (j) Comparative topography (AFM) of mold and resultant CNC film. (k) AFM line profile analysis demonstrating high-fidelity pattern transfer between mold (black) and CNC film (red) corresponding to lines in (j).

We next assessed the pattern fidelity. The parametric studies in Figure 2f–i systematically evaluate amplitude fidelity as a function of concentration, temperature, time, and pressure. Employing a mold with pattern periodicity of 2 μm and amplitude 566 nm, concentrations below 8 wt % were found to yield poor fidelity, probably due to excessive water vapor preventing CNC films from establishing conformal contact within the grooves.^36^ Given the limited impact of temperature and time on fidelity, lower temperatures and shorter processing times were generally employed. The applied pressure exhibited a threshold, where <10 kPa exhibited reduced pattern fidelity, and higher pressures yielded consistent nanopattern replication. A combination of process parameters, 8 wt % CNC suspension, 180 °C, 10 min, 10 kPa, produced a high-fidelity nanopatterned CNC film, achieving a pattern fidelity of 92%, which substantially surpasses previously reported values of approximately 50%.^36,37^ AFM images (Figure 2j) and quantitative comparison of line profiles (Figure 2k) of the nanopatterned CNC film and mold confirm excellent pattern transfer under the above conditions. To confirm the repeatability of our approach, each processing condition was independently repeated 3–6 times, and each sample was measured at 5 distinct locations, from where measurement uncertainties are estimated. AFM and microscopy data related to the pattern fidelity optimization process (Figures S13–S17) show that deviations in any single parameter can compromise pattern quality and process efficiency. Overall, wet-NIL provides an efficient and rapid process of fabricating high-fidelity nanopatterned CNC films with controlled surface topography.

### Durability of the Wet-NIL Patterned CNC Film

The hydrophilicity of nanocellulose makes nanopatterned CNC films potentially susceptible to damage by moisture,^28^ and their stability to exposure to humidity and water are critical criteria for wet-NIL imprinting. Nanopatterned CNC films were thus subjected to varying humidity and examined by small-angle light scattering (SALS), as illustrated in Figure 3a, measuring changes in pattern periodicity and amplitude (diffraction peak position *q* and corresponding intensity *I*, respectively). Upon relative humidity (between 5 and 90% RH), patterned CNC films were found to preserve their structural integrity (Figure 3b), as diffraction peak positions (first and second order) remain unchanged and only the intensity distribution changes slightly. In Figure 3c, the normalized intensity ratio (*I*_1_/*I*_max_) is shown to oscillate with the change in RH and fully recover after each RH cycle, also as evidenced by the surface morphology of the CNC films before and after humidity cycles (Figure S18). A hysteresis loop characteristic was exhibited, associated with moisture adsorption and release in CNC films (Figure 3d). Two humidity change rates (*r*), Δ*RH*/Δ*t* ≈ 1.7 and 0.9% RH/min, are represented by curves with circular and triangular markers, respectively, and greater hysteresis was observed at the higher *r*, while the lower *r* remains close to equilibrium (corresponding to a linear relation between diffracted intensity and RH).

**Figure 3:**
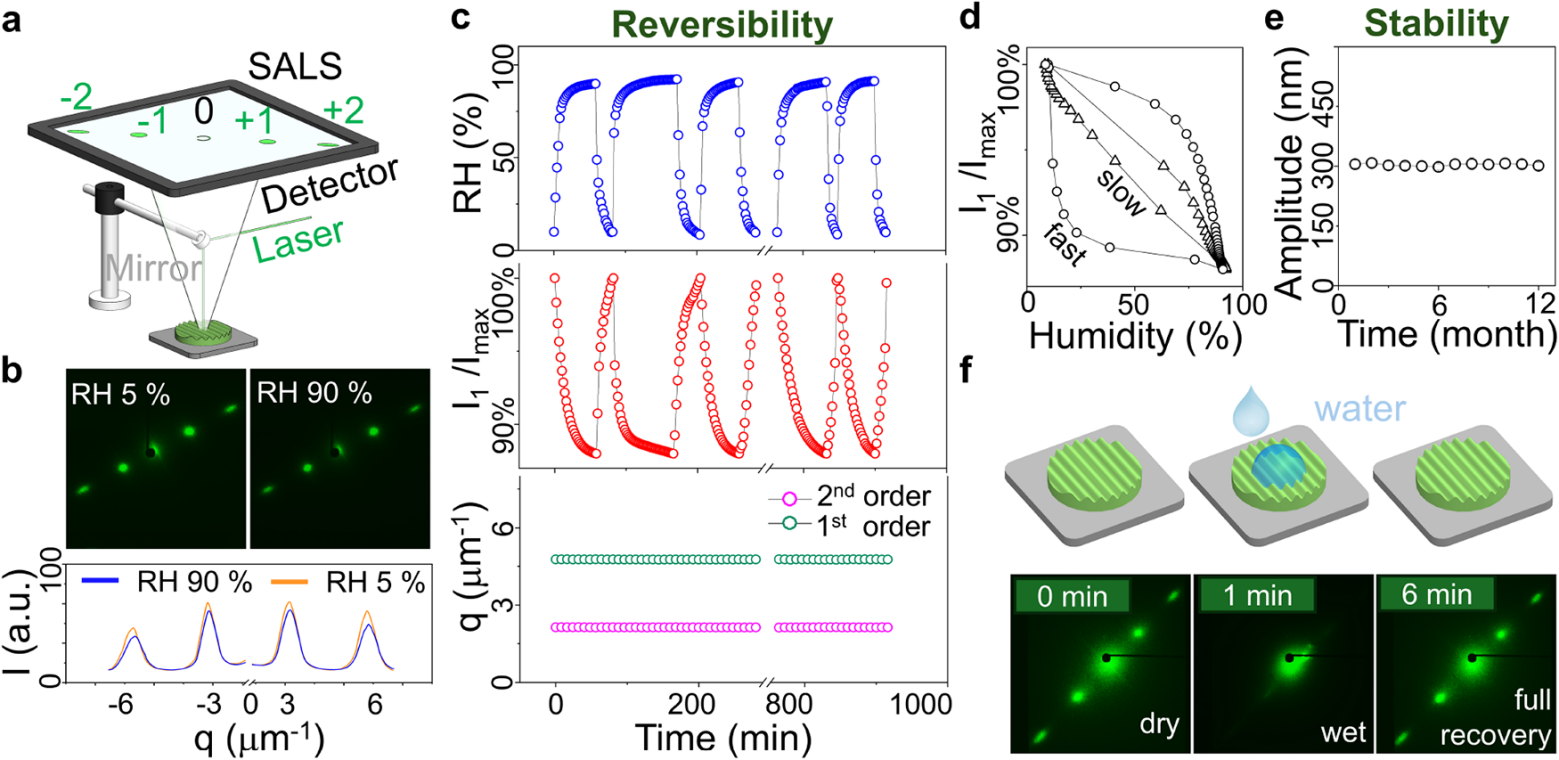
Durability of CNC nanopatterned films. (a) Schematic representation of small-angle light scattering (SALS) experimental configuration showing up to two orders (±1, ±2). (b) Comparative diffraction patterns (top) at low (5%) and high (90%) relative humidity conditions with corresponding 1st order intensity profiles (bottom). (c) Humidity cycling tests displaying RH changes (top panel) and corresponding 1st order intensity response (*I*_1_/*I*_max_) (middle panel) with consistent q-values over time (bottom panel). (d) Normalized intensity–humidity response curves at varying humidity change rates (triangle: slow, circle: fast). (e) Long-term structural stability analysis over a 12-month evaluation period. (f) Schematic and SALS illustration of dynamic water responsiveness showing rapid and complete structural recovery after a water droplet (2 μL) wetting-drying cycle.

In fact, the nanopatterned CNC films exhibit exceptional long-term stability of the pattern amplitude over 12 months (Figure 3e). Further, patterns exhibit full recovery after direct water exposure (2 μL droplet), as shown in Figure 3f and Video S1, recovering the original diffraction pattern within 6 min of drying (at least after 10 cycles, shown in Figures S19 and S20). These confirm the reversibility and structural stability of nanopatterned CNC films to RH cycles and water microdroplet contact, as required for practical applications in surfaces and coatings. Partial structural reversibility was observed upon contact with large (100 μL) water droplets over prolonged times (6 h), with the degree of recovery strongly depending on duration of exposure and water volume (Figure S21).

### Structural Colors from Nano- to Microscale CNC Patterns

Patterned CNC films act as surface gratings and can generate reflective and diffractive structural color. While the chiral assembly of (planar) CNC films has been widely reported for its intrinsic structural color, grating diffraction can augment optical effects in terms of color selection and vibrancy.^38–40^ We investigated tunable single-color selection by systematically varying the periodicity of the CNC grating as a function of viewing angle (schematically shown in Figure 4a). The relationship between observed light wavelength and viewing angle is readily described in terms of a surface grating, *n*λ = *d* sin θ, where *n* is the diffraction order (integer), λ is the wavelength of incident light, and θ is the viewing angle,^54,55^ for incident white light incident normal to the surface.

**Figure 4:**
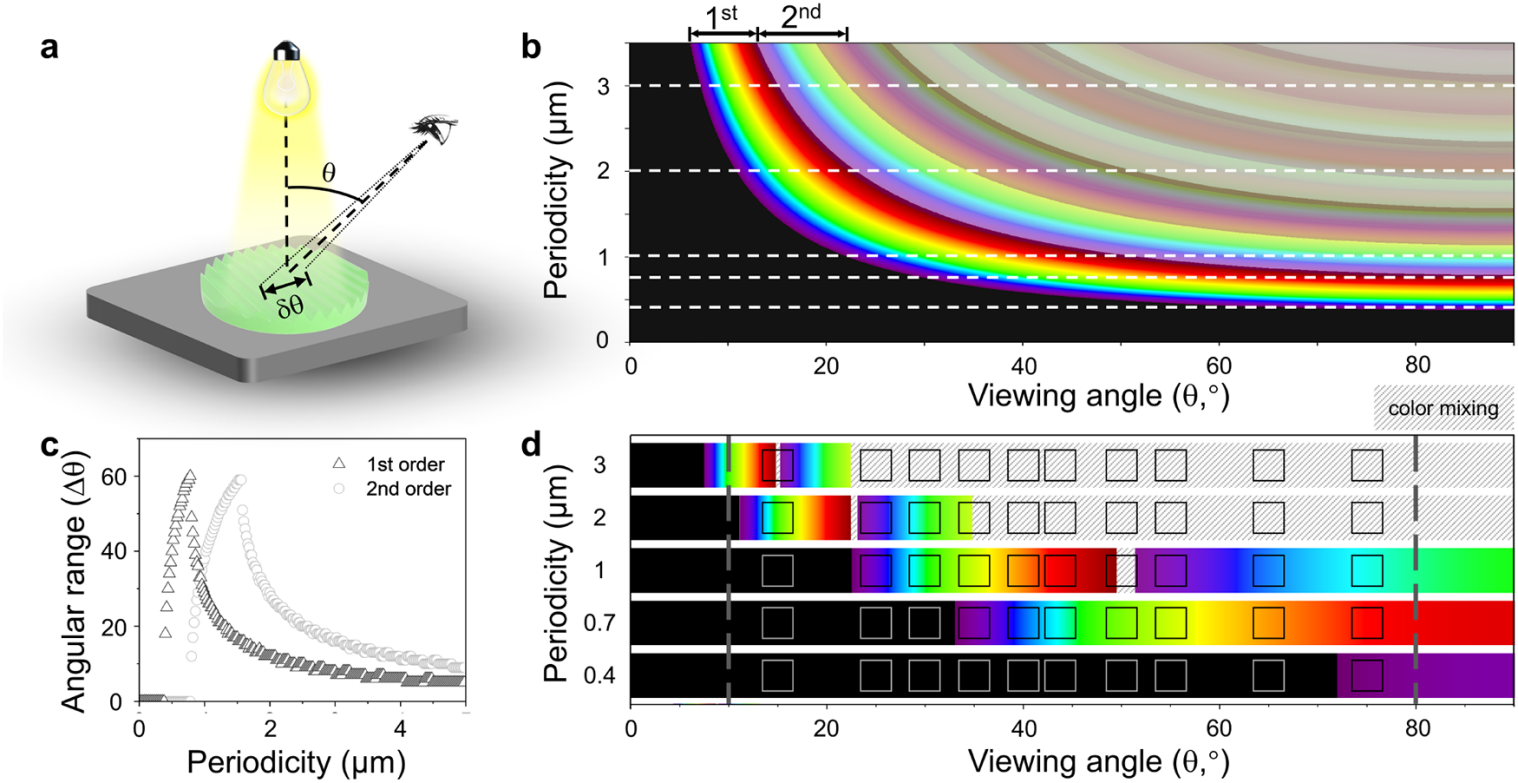
Modeling of angle-dependent structural color from periodic surface patterns. (a) Schematic illustration of experimental setup showing viewing angle (θ) and visual angle range (δθ, corresponding to the white box in (f), ±3°). (b) Theoretical dispersion diagram mapping periodicity (0–3 μm) versus viewing angle (0–90°) for 7 diffraction orders. (c) Quantitative relationship between angular range (Δθ) of single color and periodicity (μm) for first and second diffraction orders. (d) Single-color distribution map of the first order as a function of viewing angle and surface periodicity, corresponding to the white lines in ([Fig fig4]b).

Calculated color dispersion correlating periodicity *d* (within 0–3 μm) with viewing angle (0–90°) for multiple diffraction orders is presented in Figure 4b, where the first and second orders are labeled. Here, for visual representation, the wavelengths of light corresponding to specific diffraction orders at given angles and grating periodicities were converted to RGB values. The black regions in Figure 4b, corresponding to the cases where the grating periodicity is shorter than the wavelength of visible light (e.g., angles <20° and periodicity <1 μm), indicate that there is no visible light diffraction (shifting instead to UV and X-ray diffraction ranges). At higher θ and *d*, multiple diffraction orders emerge and overlap, causing color mixing from the simultaneous contribution of different diffraction orders at given viewing angles for a fixed grating periodicity.

Figure 4c illustrates the calculated angular range (Δθ) for which a *s*ingle color is observed, for both 1st and 2nd diffraction orders of a surface grating, as a function of pattern periodicity. Such an angular range is depicted by the intersection of the horizontal dashed lines and the color map in Figure 4b. In order to maximize the angular viewing range for CNC film color, we thus aim for a grating periodicity of approximately 1 μm. Beyond the second order, the Δθ available for observing a single color becomes increasingly limited. Longer periodicities yield a larger angular range for color, which, however, becomes dominated by mixed colors. Figure 4d provides a single color distribution map as a function of viewing angle and periodicity, with the color strips corresponding to the white dotted lines depicted in Figure 4b. The discrete color regions indicate areas where pure colors can be observed, while the hatched regions correspond to zones with mixed colors. The square boxes illustrate the viewing angular range of an observer (estimated at ≈3°).

The practical realization of these estimations is demonstrated through the CNC gratings (Figure 5a) with varying periodicity (0.4–3 μm), further detailed AFM images and SEM images are shown in Table S4, Figures S22 and S23. To enhance the visibility of a structural color from the CNC gratings, we dope the CNC suspension with carbon black (0.1 wt %). Figure 5b presents the photographic matrix depicting the actual structural color observed from these films as a function of the viewing angle and periodicity. Vivid single colors with the largest viewing range were observed at a periodicity of 1 μm, as expected from the estimations of Figure 4c,d. The systematic color progression closely follows the theoretical predictions (individual images correspond to squares in Figure 4d). Smaller periodicity (0.7–3 μm) requires larger viewing angles, and the full color spectrum of colors shifts with viewing angle, while larger periodicities (2–3 μm) exhibit more complex optical responses due to contributions from multiple diffraction orders, resulting in mixed colors. To quantify the color purity of the nanopatterned CNC films, we mapped the observed colors onto the Commission Internationale de L’Eclairage (CIE) 1931 chromaticity diagram (Figure 5c). For the optimal periodicity of 1 μm, the colors span a broad region of the visible spectrum as the viewing angle varies from 25° to 62°, demonstrating excellent color tunability.^56,57^

**Figure 5:**
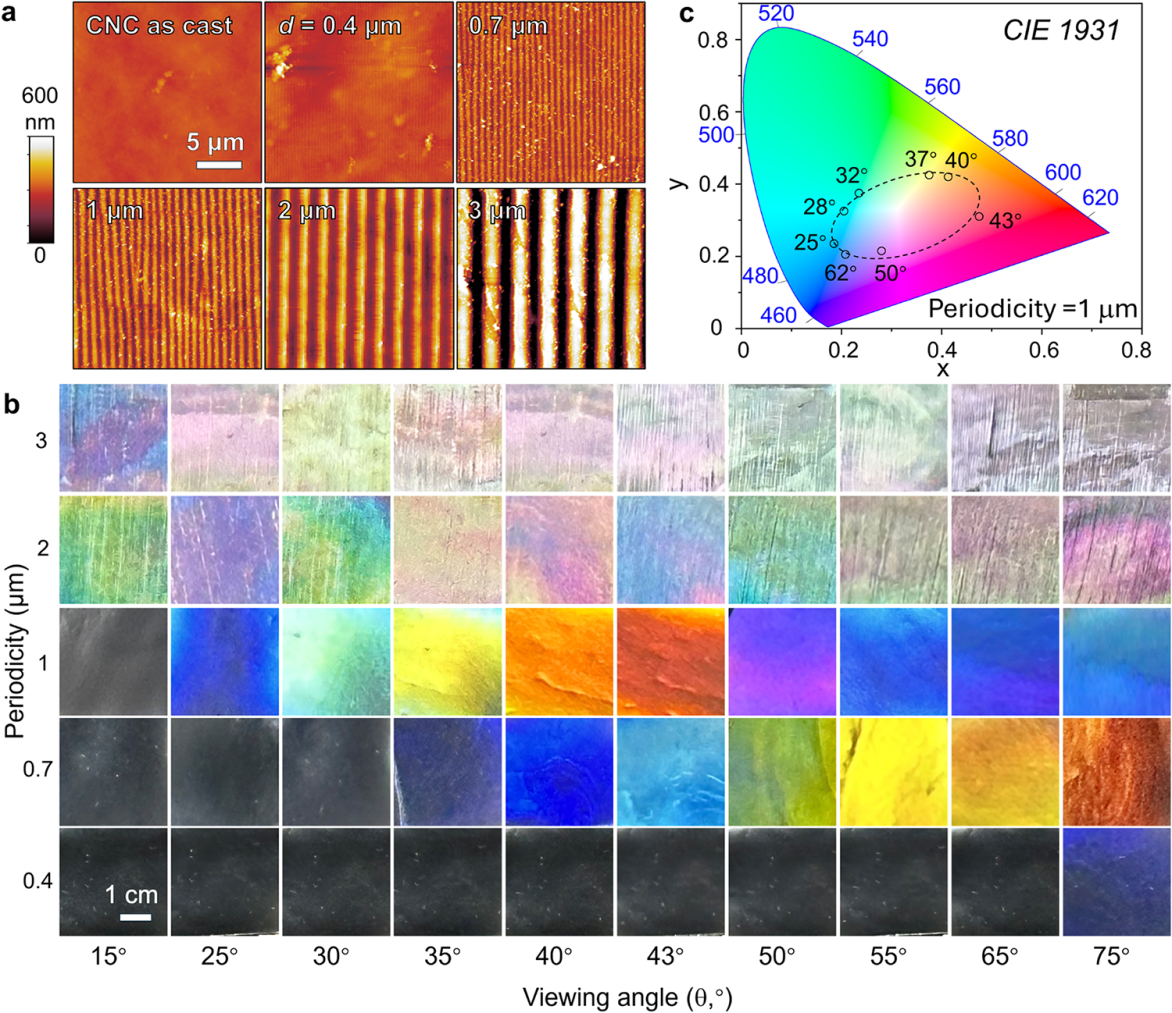
Angle-dependent structural color from nanopatterned CNC surface. (a) AFM images of nanopatterned CNC films with varying periodicities (0.4–3 μm). (b) Photographic matrix demonstrating actual structural coloration as a function of viewing angle (15–75°) and periodicity (0.4–3 μm). (c) CIE 1931 chromaticity diagram showing the color gamut achieved by CNC films with 1 μm periodicity at different viewing angles (25–62°).

### Color Vibrancy Control of the Nanopatterned CNC Film

Precise control of the pattern amplitude is required to achieve vibrant nanopatterned CNC films. Diffraction intensities for colored wrinkled structures can be estimated according to refs 58,59 .

3 $$I\approx \sum_{n=-\infty }^{\infty }J_{n}^{2}\left(\frac{m}{2}\right){\mathrm{sinc}}^{2}\left[\frac{W}{\pi }\left(q-\frac{2p\pi }{d}\right)\right]$$

where *J*_*n*_ is a Bessel function of the first kind, *W* is the half-width of the aperture (≈0.5 mm in our measurements), *d* is the wrinkling periodicity, and *n* is an index accounting for the *n*th diffraction order. The maximum intensity corresponding to the *n*th order can be approximated to be proportional to the Bessel function *J*_*n*_^2^(*m*/2), as the sinc function is narrowly distributed in *m* about each order without significant overlap between adjacent orders. The phase contrast, *m*, can be rationalized in terms of the wrinkling bilayer model of amplitude, *A*, by

4 $$\frac{m}{2}=2\pi \frac{A}{\lambda }\left[p-1\right]$$

where *A* is extracted from the wrinkling high deformation model (eq 2), *p* is the refractive index, and λ is the light wavelength.

The theoretical dependence of diffraction intensities with different amplitudes for light wavelengths of 532 nm (380 and 780 nm in Figure S24) is shown in Figure 6a, predicting that the highest vibrancy occurs within a specific amplitude range (approximately 250–400 nm), suggesting an optimal amplitude value for structural color brilliance. Figure 6c shows the surface profiles for samples i-v, showing a systematic variation in the wrinkling amplitude (ranging from 250 to 520 nm) at a constant periodicity (2 μm). The experimental measurements show excellent agreement with this theoretical prediction, as demonstrated by the SALS diffraction intensity data (green triangles in Figure 6a) and the corresponding reflectance spectra (Figure 6b). The reflectance intensity exhibits a characteristic peak around 460 nm, consistent with the diffraction behavior of periodic surface relief structures with a periodicity of 2 μm.^60^ Pattern surface roughness is expected to broaden the diffraction profile peaks (reduces peak intensity and sharpness) but does not significantly shift the spectral position. In this framework, the refractive index (*p*) of the film impacts primarily the relative intensity of the various diffraction orders, via eqs 3 and 4, and changes in *p* (e.g., due to additives or residual water) can modulate the diffractive color intensities. Here, we have taken *p* ≃ 1.54 for CNC films and, given the relatively low roughness (RMS ∼ 20–30 nm, <5–10% height) and 92% pattern fidelity, deviations from model assumptions are relatively minor.

**Figure 6:**
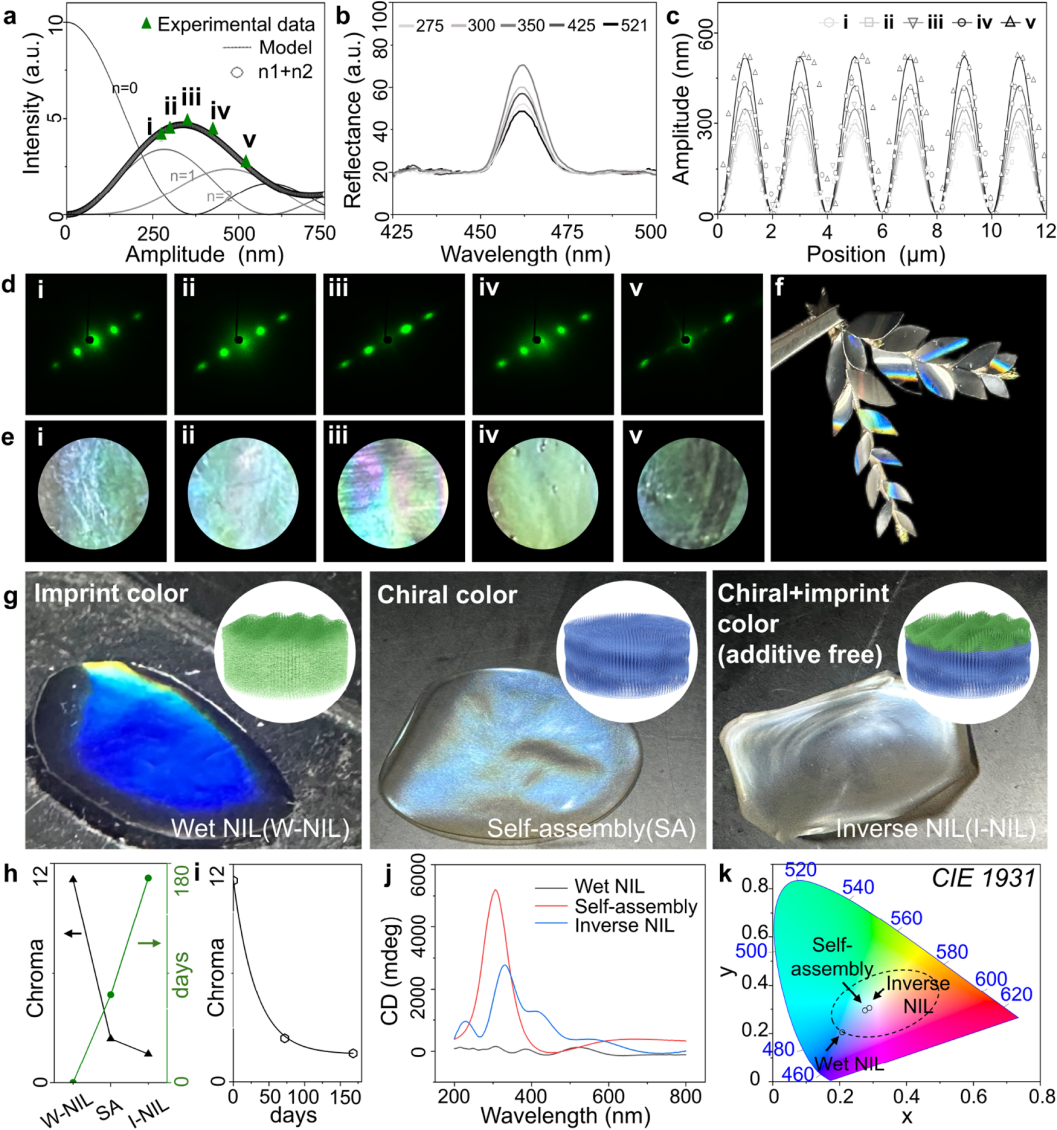
Amplitude-dependent color vibrancy control and structural coloration method comparison. (a) Quantitative intensity distribution analysis of the theoretical model and different amplitude patterns (i–v) with experimental data (green triangles). (b) Reflectance spectra of nanopatterned CNC surface with varied amplitudes. (c) AFM height profiles of the five pattern amplitudes (i–v). (d) SALS diffraction patterns and (e) corresponding optical images from CNC films with increasing pattern amplitudes (i–v). (f) Photo of iridescent fake leaves made of nanopatterned CNC films. (g) Comparison of color generation mechanisms in CNC films: imprint-based reflective structural coloration (left), intrinsic chiral coloration (middle), and additive-free combined coloration effect (right) with corresponding microstructure schematics (insets), showcasing the strength of the wet-NIL method for tunable optical properties. (h) Chroma and manufacturing time comparison between the three fabrication methods. (i) Correlation between manufacturing time and chroma of three fabrication methods. (j) Circular dichroism (CD) spectra of structural colored samples from the three methods. (k) CIE 1931 chromaticity diagram showing the deep blue color gamut achieved by each method.

Figure 6d shows that the SALS diffraction patterns varied with the wrinkling amplitude; the corresponding visual appearances under omnidirectional illumination displayed different brightness in Figure 6e. Combining the tunable color and controllable vibrancy, a demonstration of leaf-shaped fragments is shown in Figure 6f.

### Benchmarking Color Achieved by Wet-NIL, Chiral Assembly, or Hybrid Methods

Finally, a comparison of vibrant blue coloration achieved via a wet-NIL, chiral self-assembly, and inverse NIL was conducted. These three methods rely on distinct structural coloration mechanisms: (1) Wet-NIL generates color purely from imprinted surface relief structures; (2) Chiral self-assembly derives color from the intrinsic chiral nematic ordering within the CNC matrix; and (3) inverse NIL combines both chiral and imprint-induced structural features. Schematic illustrations of each approach are shown in the inset of Figure 6g. A quantitative comparison of the three methods reveals their performance trade-offs. Figure 5h and *i* shows that wet-NIL achieves the highest chroma (≈11) with the shortest fabrication times (∼10 min), representing a 100–160 fold time reduction compared to chiral self-assembly (7 days) and inverse NIL (3 days). While inverse NIL can potentially offer the most vibrant colors and iridescence due to its dual structural contributions, it yields the lowest chroma (≈1.6) due to film deformation and compromised structural integrity for pure CNC composition, i.e., without film-forming additives. Circular dichroism (CD) spectra (Figure 6j) reveal that, as expected, slow evaporation-driven self-assembly produces the strongest chiral nematic order, inverse NIL partially preserves this chiroptical response, both with peak intensities around 400–500 nm. Whereas direct wet-NIL largely erases the cholesteric organization, only residual molecular-level chirality remains, yielding almost CD-silent films whose coloration is dominated by nonchiral surface structuring.^61,62^ In the CIE map (Figure 6k), films produced by wet-NIL exhibit vibrant and angle-independent blue color with the highest color purity among the three methods for pure CNC films.

## Conclusions

We demonstrate the fabrication of nanopatterned CNC films via a nanoimprinting lithography approach, which we term wet-NIL, employing a semipermeable polymeric mesh support that permits water removal and film formation during pattern transfer over min time scales. Wet-NIL is found to be well suited for CNC film patterning and, as expected, is less applicable to BNC and CNF pattern transfer at nanoscale mold dimensions. The wet-NIL bridges thus nano-micro length scales (from 200 nm ≤ periodicity ≤7 μm and from 275 nm ≤ amplitudes ≤ 520 nm), within a range customarily included within “nanoimprint lithography” in the literature. The optimization of process parameters, including CNC concentration and rheology, imprint temperature, pressure, and time, has enabled high-fidelity (92%) replication and short patterning times, ∼ 10 min (employing 8 wt % CNC, 180 °C, and 10 kPa) across nano- and microscales. By tuning the wavelength and amplitude of CNC surface patterns, we obtain diffractive structural color, with precise and quantitative control over color, vibrancy, and angular range, in quantitative agreement with phase grating optics modeling.

While evaporation (or solvent loss) of water from CNC dispersions is well-known to lead to the formation of a chiral nematic phase, which can exhibit structural color by adjusting the pitch of the helicoidal into the visible range, this process is inherently slow due to self-assembly, viscosity increase, and eventual kinetic arrest.^17^ Typically, CNC colored films are formed by slow evaporation over several days to weeks (while fast evaporation results in disordered noncolored films). By contrast, we demonstrate that the wet-NIL process can reduce time scales to obtain structurally colored down to 10 min, or 3–4 orders of magnitude faster. Further, color can be readily and predictively tuned by the imprint mold (without resorting to chemical modification, solvent exchanges, or blending). Surface patterning and cholesteric self-assembly of CNC can be combined^38,39^ to yield a “dual” photonic structure, often aided by a film-forming water-soluble polymer (such as poly­(vinyl alcohol), PVA) to ensure planarity, or solvent additives to tune color response. However, the fabrication time scales associated with such nanoimprinting lithography processes accompanied by evaporation-induced self-assembly (*v*iz. drying a CNC suspension onto a diffraction grating profile, sometimes referred to as inverse NIL) are governed by the same long time scales of self-organization.

A range of approaches are shown to be compatible with the wet-NIL fabrication of CNC films. We demonstrate mold fabrication by surface wrinkling of PDMS via plasma exposure and uniaxial strain relaxation to generate the tunable nanoimprint molds (and thus tunable color response), replicated onto thiolene supports. Further, we show that nanopatterned CNC films obtained by wet-NIL exhibit excellent environmental stability, maintaining structural integrity and reversible topographic changes (∼10%) through humidity cycling and direct water droplet exposure.

By leveraging the intrinsic properties of CNCs alongside facile surface patterning, we report sustainable, biodegradable, cellulose-based coatings with versatile optical functionality, within minute time scales. This scalable and rapid CNC nanofabrication film technique represents a promising, viable alternative to conventional synthetic polymer or inorganic materials in various technologies where sustainability and photonic functionality are both required, including in security features, coating, and sensing applications.

## Methods

### Materials

CNCs were kindly supplied by Melodea Ltd. Poly­(dimethylsiloxane) (PDMS, Sylgard 184, Dow Corning) was purchased from the DOW Chemical Company. Norland optical adhesive 81 (NOA 81) was obtained from Edmund Optics. Carbon black acetylene powder (50% compressed, >99.9%) was purchased from VWR International, LLC. Poly­(vinylidene fluoride) (PVDF) mesh was purchased from Merck. All chemicals were used as received without any further modifications.

### CNC Characterization

The morphology and crystallinity of CNC were characterized by transmission electron microscopy (TEM), dynamic light scattering (DLS), and wide-angle X-ray scattering (WAXS). WAXS measurements were performed using a Xenocs Xeuss 2.0 C instrument with a Cu Kα X-ray source (30 W). The crystallinity index was then determined using the Ruland method^63^ according to the following equation:

$$\chi_{c}=\frac{\int_{q_{0}}^{q_{1}}I_{c}q^{2}dq}{\int_{q_{0}}^{q_{1}}Iq^{2}dq}$$

where *I*_c_ is the intensity of the cellulose *I*_β_ scattering profile, *I* is the total calculated scattering intensity (sum of calculated crystalline and amorphous contributions), *q* is the scattering vector given by 4π sin θ/λ, and the integration limits *q*_0_ and *q*_1_ are 0.36 and 3.83 Å^–1^, respectively. The details of measurement and data analysis are shown in Figure S2. The density of carboxyl groups was determined through conductometric titration (Figure S3). The rheological behavior of CNC suspensions was examined using an Anton Paar MCR 302 rheometer equipped with parallel plates (20 mm plate, 500 μm gap) at a controlled temperature of 25 °C. Steady shear sweeps were performed over a shear rate ranging from 0.01 to 2000 s^–1^ to monitor the apparent viscosity. ζ-Potential was measured with a Malvern Zetasizer MicroV apparatus; details are in Supporting Information (SI).

### Mold Preparation

PDMS was prepared with a 10:1 mass ratio of prepolymer to cross-linker, stirred thoroughly, and degassed under vacuum. The mixture was then poured into a plastic Petri dish to achieve a thickness of 2 mm for the 1D samples. It was then cured at 75 °C for 1 h in a convection oven and subsequently cut into coupons measuring 6 cm × 5.5 cm. Wrinkled surfaces were generated by oxygen plasma oxidation under strain, as illustrated in Figure 1f, employing two distinct plasma chambers: for submicron wrinkle periodicity, a 13.6 MHz Harrick Plasma PDC-002 system was operated at 10.5 W; while for samples with larger wrinkling periodicity, a 40 kHz Diener Plasma (Femto) system, equipped with a TM 101 Thermovac pressure sensor, was used at 20, 50, and 99 W with variable exposure times. In both systems, oxygen (BOC, 99.5%) was supplied. The chambers were evacuated to 0.2 mbar, followed by 5 min of gas flow to achieve the desired pressure. Plasma was then exposed at the specified power levels for a specific duration.

One-dimensional (1D) linear patterns were formed by applying uniaxial strain to a PDMS coupon by using a strain stage. The prestrain $\epsilon =\frac{L_{1}-L_{0}}{L_{0}}$ , where length (*L*_0_) is the original length and *L*_1_ is the final length of PDMS after stretching. Varied prestrains were applied to the PDMS, followed by plasma oxidation; detailed strain information is shown in Table S1. Upon releasing the strain, the sinusoidal wrinkles were simultaneously generated on the surface of the sinusoidal line profile (Figure S10).

Two-dimensional (2D) chevron wrinkling patterns were fabricated by applying a simultaneous biaxial strain of ϵ = 0.1 to a PDMS coupon, followed by plasma treatment at 20 W for 2 min. The strain was then slowly and simultaneously released, to minimize crack formation,^64,65^ resulting in the formation of the chevron pattern.

Isotropic samples were prepared via plasma oxidation of a thin PDMS film.^65,66^ PDMS was spin-coated onto 1 cm^2^ silicon wafers to form an 11 μm-thick film, then cured at 75 °C for 1 h. The coupons were plasma-treated at 99 W for 5 min, leading to wrinkle formation upon removal from the plasma chamber.

The PET–PDMS mold was fabricated using nanoimprint lithography (NIL) with an EVG 620 semiautomated nanoimprint lithography system, equipped with Smart NIL tooling for high-precision UV-based nanoimprinting. The 1.5 μm pitch 4-in. hexagonal mask for NIL was fabricated by Displacement Talbot Lithography (DTL) with a PhableR 100 M system from EULITHA AG (detailed in Figure S6).

PDMS wrinkled surfaces were replicated onto NOA 81 by pouring uncured resin onto the PDMS mold and securing it with a hollow PMMA frame. A UV ozone system (PSD-UV, Novascan) was utilized to cure the NOA resin for 1 h.

### Wet-NIL Process

The CNC suspensions (initial concentration of 5 wt %) were either concentrated or diluted to concentrations varying from 4 to 12 wt %. A total of 0.06 g (dry weight) CNCs was deposited onto a semipermeable support membrane, which serves as a substrate, and was fixed by hollow metal frames. The CNC suspension was subsequently compressed by using a nanopatterned mold under controlled pressure (0–100 kPa), forming a wet CNC cake. This assembly was then subjected to thermal treatment at temperatures ranging from 20 to 220 °C, facilitating water evaporation and structural consolidation. Upon the removal of the mold following complete drying, a free-standing nanopatterned CNC film (65 g/m^2^) was obtained. Every processing condition was repeated 3 to 6 times, and each sample was measured in five distinct regions. 0.1 wt % carbon black was added to the CNC suspension and thoroughly dispersed using an Ultra-Turrax homogenizer (T25, IKA) to enhance the visibility.

### CNC Self-Assembly Process

An evaporation process was used to achieve a chiral self-assembled CNC film. CNCs were first dispersed in deionized water to create a 0.1 wt % aqueous suspension. The suspension was briefly sonicated (100 W, 10 min) to ensure a uniform dispersion and then centrifuged at 5000 rpm for 15 min to remove larger aggregates. The supernatant was then carefully transferred to a clean glass Petri dish. Then the suspension was allowed to evaporate under controlled conditions (23 ± 2 °C, 50 ± 5% relative humidity) in a dust-free environment. After 7 days of slow drying, a CNC film (65 g/m^2^) with chiral helicoidal nanostructure was formed.

### Inverse NIL Process

An evaporation-based method was also used to achieve the bulk chiral and surface-patterned CNC films. A PDMS mold with λ of 2 μm and *A* of 566 nm was selected. Before the CNC suspension was transferred, the PDMS mold was sandwiched by metal frames and treated with oxygen plasma for 20 s. Then the CNC suspension (0.06 g dry weight, 5.0 wt %) was transferred onto the PDMS mold. This mixture was allowed to evaporate under controlled conditions (23 ± 2 °C, 50 ± 5% relative humidity) in a dust-free environment (for 3 days). Subsequently, the CNC film (65 g/m^2^) was obtained.

### Small-Angle Light Scattering Measurement

Static light scattering measurements were performed using a custom-built setup consisting of a diode-pumped Crystal Laser (λ = 532 nm, power = 500 mW) with laser light directed perpendicular to the sample surface and reflected by a mirror at 45°. The laser intensity was controlled using neutral density (ND) filters (from Thor Laboratories), which optimizes signal detection without camera saturation. Scattered light patterns were captured using a CCD camera (Hamamatsu Orca), controlled by the in-built software Wasabi. The data were analyzed with Fiji software. Various relative humidity conditions (5–90% RH) were adjusted in a homemade humidity chamber constructed from poly­(methyl methacrylate) (PMMA). Water (2 μL) drops were then directly placed on the sample surface using a Hamilton microsyringe.

### Pattern Characterization

Surface topographies of PDMS molds and nanopatterned CNC films were characterized using a Bruker Innova atomic force microscopy (AFM) operating in tapping mode, equipped with Al-coated silicon tips (MPP-11100-W, Bruker). Surface topographies of NOA molds were characterized using a Jupiter XR large-sample AFM equipped with ultrasharp silicon tips (tip radius <10 nm) in tapping mode. Data analysis was performed using the Gwyddion software.

The mechanochromic phenomena were observed and recorded under white light illumination (Advanced Illumination) in a dark environment. The recorded images were subsequently analyzed with the Fiji software. Commission Internationale de L’Eclairage (CIE) chromatic coordinates, chroma, and reflection spectra were measured via a stella black comet C spectrometer (StellaNet). Circular dichroism spectra were recorded using a Chirasacan CD spectrometer (Applied Photophysics).

## Supplementary Material





## Notes

Detailed characterization data and methodological details, including: nanocellulose characterization (TEM, WAXS, conductometric titration, ζ-potential measurements, and morphological analysis of CNF and BNC); evaluation of semipermeable support membranes for the wet-NIL process; mold design and manufacturing procedures (including PDMS and NOA molds fabricated via displacement Talbot lithography and wrinkling methods); systematic investigation of wet-NIL processing parameters (concentration, temperature, time, and pressure effects on pattern fidelity); stability testing under humidity and water exposure conditions; comprehensive morphological characterization of patterned CNC films with varying periodicities (0.2–7 μm) using AFM and SEM; and theoretical modeling of diffraction intensity as a function of surface amplitude and wavelength (PDF)
Patterns exhibit full recovery after direct water droplet exposure,illustrated with a 1D surface of 10 × 10 mm^2^ and 2 μm periodicity, onto which a 2 μL deionized water droplet is placed at ambient temperature and ∼40% RH (Video S1) (MP4)
