# Supplementary material for: Rapid Nanocellulose Wet Nanoimprint Lithography for Tunable Structural Color
Source: ACS Nano. 2025 Dec 23;20(1):823–34. doi: 10.1021/acsnano.5c15904 (PMC12810477; doi:10.1021/acsnano.5c15904)
Supplement: Supplementary file 1 [file nn5c15904_si_001.pdf]

# Supplementary Information:

## Rapid nanocellulose wet nanoimprint lithography for tunable structural color

Hui Mao,[a] Zain Ahmad,[a] Lu Xin,[a] Hisay Lama,[a] Pengfei Fan,[b] Philip Shields,[b] Samuel Eyley,[c] Wim Thielemans,[c] João T. Cabral[a]*

[a] *Department of Chemical Engineering, Imperial College London, London, SW7 2AZ, United Kingdom*

[b] *Centre for Nanoscience and Nanotechnology, University of Bath, Bath, BA2 7AY, United Kingdom*

[c] *Sustainable Materials Lab, Department of Chemical Engineering, KU Leuven Kulak Kortrijk Campus, E. Sabbelaan 53, 8500 Kortrijk, Belgium*

***Email:** j.cabral@imperial.ac.uk

# 1 Nanocellulose Characterization

Nanocellulose (NC) exists in several forms, each with distinct properties suited for different applications. Cellulose nanocrystals (CNCs) are rod-like nanoparticles (5-20 nm width, 100-500 nm length) with high crystallinity, typically produced through acid hydrolysis of cellulose fibers.[1,2] Cellulose nanofibrils (CNFs) are longer, more flexible fibrils (5-50 nm width, several micrometers length) produced through mechanical fibrillation, often preceded by chemical or enzymatic pretreatment.[3] Bacterial nanocellulose (BNC) is produced extracellularly by certain bacteria, resulting in highly pure nanofibrils with exceptional mechanical properties and water-holding capacity.[4] Among these, CNCs are particularly attractive for photonic applications due to the real nano-scale size in both width and length and their ability to self-organize into ordered structures.[5]

Firstly, CNCs were characterized using transmission electron microscopy (TEM, courtesy of Y. Nevo, Melodea) and Wide-angle X-ray scattering (WAXS), providing detailed information on their morphology and crystallinity. In practical term, electrostatic stability of NC suspension plays a vital role for its shelf life. The carboxyl group density measured by conductive titration and zeta potential assessed the electrostatic stability.[6]

**TEM analysis of CNCs.**

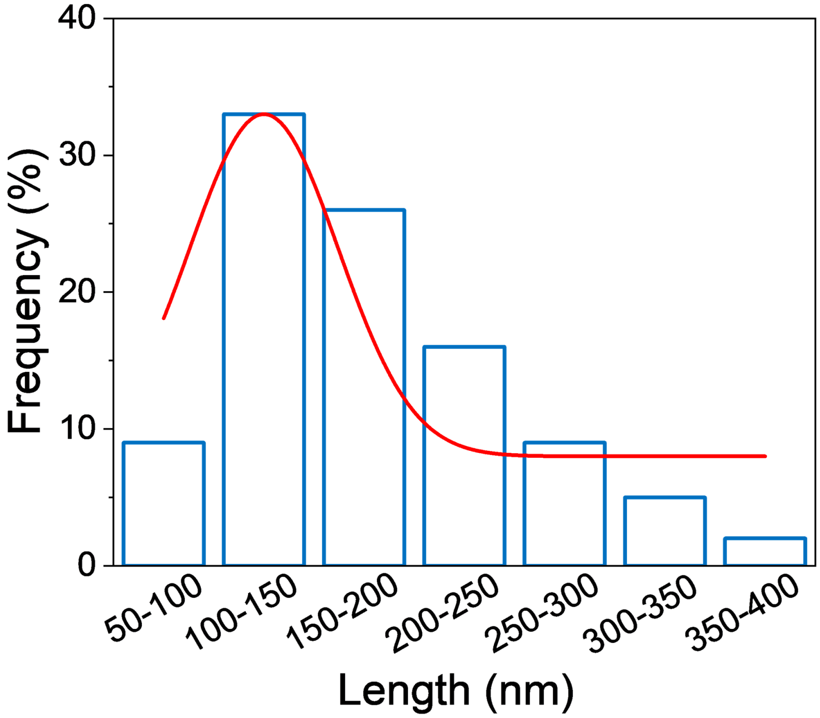


**Figure S1** Length distribution of CNC nanorods estimated from TEM.

TEM analysis was conducted to characterize the size distribution of the CNCs. The length measurements, derived from statistical analysis of over 200 individual CNC particles, revealed that more than 50% of the CNCs fall within the length range of 100–200 nm. The average length was determined to be approximately 176 nm,

indicating a relatively narrow size distribution. This consistent morphology is critical for achieving uniform behavior in subsequent applications, such as nanopatterning or self-assembly. The nanometer-scale dimensions also support strong colloidal stability and effective interaction with light in optical applications.[7] Representative TEM images and the corresponding histogram of length distribution are presented in the main text.

**WAXS measurement.**

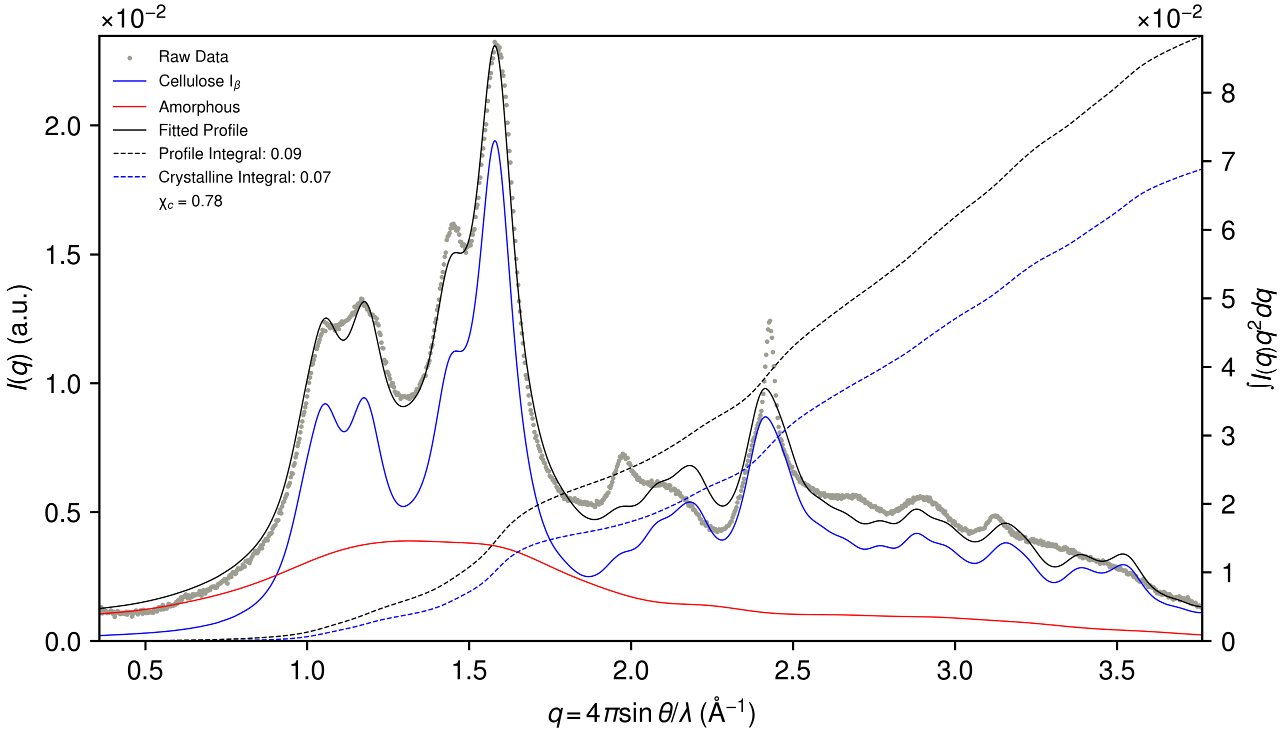


**Figure S2** X-ray scattering pattern of CNCs showing the calculated cellulose $I\beta$ and amorphous contributions to the profile

Wide-angle X-ray scattering (WAXS) measurements were performed on a Xenocs Xeuss 2.0C laboratory beamline in transmission geometry, using a copper K$\alpha$ X-ray source (1.54189 Å) with a point-collimated beam (1 mm diameter at the sample position). Scattered X-rays were detected using a Dectris Eiger 1M 2D hybrid photon-counting detector. The detector was moved through nine positions (three rows of three) perpendicular to the beam and the resulting images were stitched together to increase the accessible q-range (with q the scattering vector). The sample to detector distance (and therefore q vector) was calibrated using lanthanum hexaboride (NIST SRM660c). Data was collected with the entire beam path under vacuum (<0.1 kPa) during acquisition to reduce air scattering. The resulting 2D detector images were azimuthally integrated to give 1D scattering curves. The intensity was corrected for transmission and converted to absolute intensity using a calculated linear absorption coefficient of 11.8 cm$^{-1}$. The background curve (empty beam) was subtracted from the sample curves prior to further processing. The crystallinity index was calculated by Rietveld refinement (using TOPAS-academic v6) of two phases consisting of the cellulose I$\beta$ phase published by Nishiyama et al.[8]

and an amorphous phase measured from lignin sulfate. The phases were fitted to the data, allowing only small changes in unit cell parameters for the cellulose structure, a second order spherical harmonics function to account for preferred orientation, and a flat background to account for possible errors in instrumental background subtraction. The fitting parameters were constrained to obtain a physically meaningful fit at the expense of a poorer mathematical fit to the data. The crystallinity index was then determined using the Ruland method[9] according to the following equation:

$$\chi_c = \frac{\int_{q_0}^{q_1} I_c(q) q^2 \, dq}{\int_{q_0}^{q_1} I(q) q^2 \, dq}$$

where $I_c$ is the intensity of the cellulose I$\beta$ scattering profile, $I$ is the total calculated scattering intensity (sum of calculated crystalline and amorphous contributions), $q$ is the scattering vector given by $4\pi \sin\theta/\lambda$ and the integration limits $q_0$ and $q_1$ are 0.36 Å$^{-1}$ and 3.83 Å$^{-1}$ respectively.

**Conductive titration**

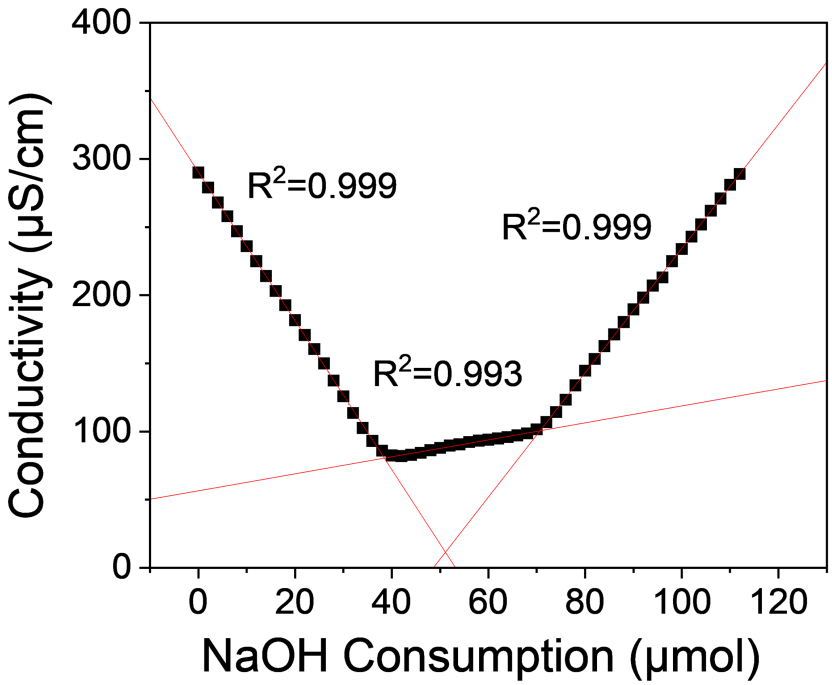


**Figure S3** Conductive titration curve of CNC suspension.

**Method**

A cellulose nanocrystal (CNC) dispersion was prepared by diluting 0.12 g (dry weight) of CNCs with deionized water to a total volume of 150 mL. To initiate cation exchange, 1 g of ion exchange resin in the hydrogen form ($H^+$) was added directly to the CNC suspension. The mixture was stirred using a magnetic stirrer at a constant speed of 500 rpm for a period of 48 hours at room temperature, allowing sufficient time for the exchange of native counterions (e.g., $Na^+$ or other metal ions) associated with the CNC surface with hydrogen ions ($H^+$) from the resin.

After the ion exchange process, a 50 mL aliquot of the treated CNC suspension was transferred to a clean container for titration analysis. To acidify the system and standardize the ionic background, 400 $\mu$L of 0.1 mol/L hydrochloric acid (HCl) was added while maintaining continuous magnetic stirring at 500 rpm. The initial conductivity of the solution was recorded under these conditions.

Subsequently, a 0.1 mol/L sodium hydroxide (NaOH) solution was added to the acidified CNC suspension in small increments of 20 $\mu$L per addition. After each addition, the solution was allowed to equilibrate briefly, and the conductivity was measured and recorded. This titration process continued until the conductivity profile indicated that the titration endpoint had been reached, reflecting the complete neutralization of available acidic groups and allowing for the characterization of the surface charge properties of the CNCs.

Carboxyl group density (D(–COOH)) of CNCs was calculated as follow:

$$D(\text{–COOH}) = \frac{n(\text{–COOH})}{0.04\,\text{g}} = \frac{n(\text{NaOH})}{0.04\,\text{g}}$$

**Zeta potential measurement**

**Method**

Dilute samples of NC suspensions with a concentration of 0.01 wt% were prepared to evaluate the zeta potential at a controlled pH of 7. The measurements were performed using a Malvern Zetasizer MicroV instrument, which employs dynamic light scattering (DLS) combined with electrophoretic light scattering to determine particle mobility and, consequently, the zeta potential. Disposable folded capillary cells were used for all measurements to ensure accuracy and avoid cross-contamination between samples.

Prior to measurement, the pH of each NC suspension was carefully adjusted to 7.00 using dilute solutions of sodium hydroxide (NaOH) and hydrochloric acid (HCl), ensuring minimal change to the ionic strength of the system. All measurements were conducted at a constant temperature of 25 °C, maintained by the instrument's internal temperature control system. For each suspension type, multiple replicates were measured to ensure statistical reliability and reproducibility.

The measured zeta potential values for the various NC suspensions were as follows: cellulose nanocrystals (CNC), $-50 \pm 3$ mV; cellulose nanofibers (CNF), $-22 \pm 1$ mV; and bacterial nanocellulose (BNC), $-21 \pm 1$ mV. These results indicate that the CNC suspension possesses the highest surface charge density, which enhances electrostatic repulsion between particles. Consequently, CNC suspensions exhibit the greatest colloidal stability and are expected to have the longest shelf life among the three types evaluated.[10]

**Morphology measurement of CNF and BNC**

Cellulose nanofibers (CNFs) and bacterial nanocellulose (BNC) were generously provided by Sappi Ltd. and Cellugy Ltd., respectively. The physical appearance, morphological characteristics, and size distributions of both nanocellulose types were comprehensively analyzed and are presented in Fig. S4. Specifically, pho-

tographs showing the macroscopic appearance of CNF and BNC suspensions are displayed in Fig. S4a and S4d, respectively. Atomic force microscopy (AFM) images, which reveal the surface morphology and nanoscale structure, are shown in Fig. S4b for CNFs and S4e for BNC. The corresponding diameter distributions, derived from statistical analysis of AFM data, are provided in Fig. S4c and S4f.

Both CNFs and BNC exhibit diameters within the nanometer range, confirming their classification as nanocellulose materials. In contrast, their lengths extend into the micrometer scale, indicating a high aspect ratio, which is typical for fibrillar nanocellulose structures. This high aspect ratio is known to contribute significantly to the mechanical reinforcement capability and network-forming behavior of nanocellulose in composite or hydrogel systems.[11,12]

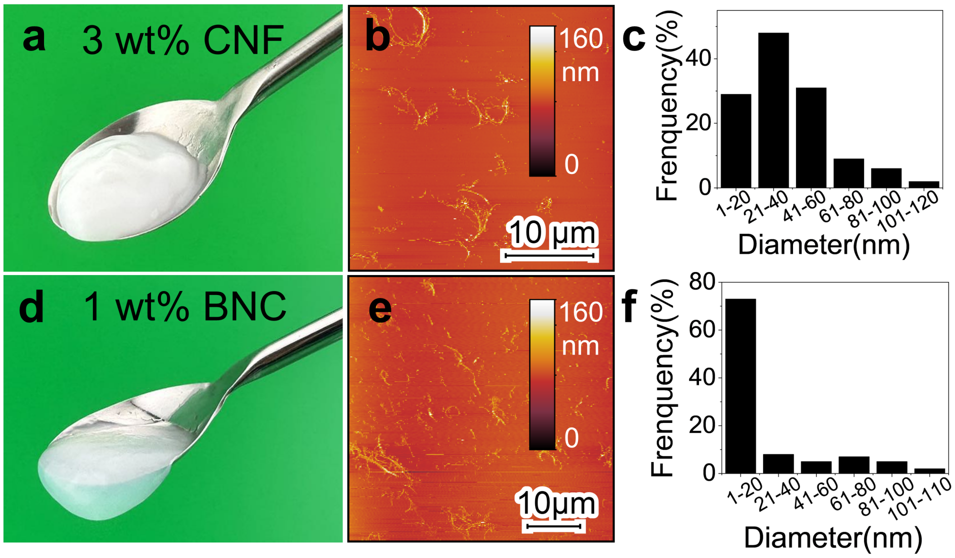


**Figure S4** Photos of CNF (a) and BNC (d) suspension. AFM images of CNF (b) and BNC (e) suspension. Diameter distribution of CNF (c) and BNC (f) suspension.

## 2 Semi-permeable support membrane evaluation

The semi-permeable support membrane plays a critical role in the wet nanoimprint lithography (wet-NIL) process, as it directly influences both the drying kinetics and processability of nanocellulose films. Key factors in the selection of a suitable support membrane include its hydrophilicity, pore size, and material composition. Considering the typical dimensions of cellulose nanocrystals (CNCs), with lengths around 176 nm, a membrane pore size of 0.22 $\mu$m is deemed optimal to achieve an appropriate balance between nanocellulose retention and water permeability. Moreover, only hydrophilic membranes permit unimpeded water passage, resist fouling by hydrophilic nanomaterials, and maintain pore accessibility without requiring additional surface treatment. Therefore, hydrophilic membrane with a pore size of 0.22 $\mu$m was selected for this study.

A comparative evaluation of commonly used membrane materials, including regenerated cellulose (RC),

nylon, polycarbonate (PC), anodic aluminum oxide (AAO), polyethersulfone (PES), glass fiber, polytetrafluoroethylene (PTFE), and polyvinylidene fluoride (PVDF), was conducted with a focus on surface smoothness, mechanical strength, and filtration efficiency. For high-fidelity nanostructure replication in wet-NIL, the membrane surface must exhibit sufficient smoothness; hence, membranes composed of nylon, PES, PTFE, and glass fiber were excluded due to their inherently rough or fibrous surfaces. Additionally, as the wet-NIL process involves the application of pressure to form nanoscale patterns, mechanical robustness is essential. Fragile membranes such as RC, PC, and AAO were therefore deemed less suitable.

Finally, filtration efficiency, which directly correlates with the processing throughput, was assessed based on water permeability data, as summarized in Table S1.

**Table S1** Summary of approximate flow rates for variable membranes

| Membrane material | Flow Rate (ml/min/cm$^2$) |
|---|---|
| Glass Fiber | 500–1000 |
| Polyethersulfone (PES) | 200–400 |
| Nylon | 100–200 |
| Regenerated cellulose (RC) | 50–100 |
| Polyvinylidene fluoride (PVDF) | 30–70 |
| Polycarbonate (PC) | 5–20 |
| polytetrafluoroethylene (PTFE) | 5–20 |
| Anodic Aluminum Oxide (AAO) | 1–10 |

After thorough evaluation, hydrophilic PVDF membranes with pore size of 0.22 $\mu$m were selected as the optimal choice for the wet-NIL process. PVDF membranes combine moderate filtration rates, mechanical durability, and a smooth surface finish, all of which are critical for achieving uniform, high-quality nanocellulose films with well-defined nanopatterns. Moreover, PVDF membranes enable easy and clean release of the films without compromising the integrity of delicate nanostructures, making them particularly well-suited for applications requiring precision and reproducibility.

# 3 Mode design and manufacturing

To demonstrate the versatility and robustness of the wet nanoimprint lithography (wet-NIL) process, a range of stamp materials with differing mechanical and surface properties were employed to imprint patterns onto cellulose nanocrystal (CNC) suspensions. Atomic force microscopy (AFM) images of the resulting imprinted patterns are shown in Fig. S5. Specifically, stamps shown in Fig. S5a and S5b were fabricated from Norland Optical Adhesive (NOA), a UV-curable polymer known for its rigidity and high pattern resolution. Fig. S5c displays patterns generated using a silicon-based stamp, which offers excellent dimensional stability and nanoscale precision. Fig. S5d and S5e present results obtained with polydimethylsiloxane (PDMS) stamps, which are widely used in soft lithography due to their flexibility, ease of fabrication, and conformal contact with substrates.

The successful transfer of nanoscale features from all five stamp types confirms the broad material compatibility of the wet-NIL process with CNC suspensions. This compatibility is particularly significant given the distinct mechanical properties and surface chemistries of NOA, silicon, and PDMS, underscoring the process's adaptability for diverse imprinting applications. The results further suggest that wet-NIL can be effectively integrated into a wide range of fabrication workflows, depending on the desired pattern characteristics and available materials.

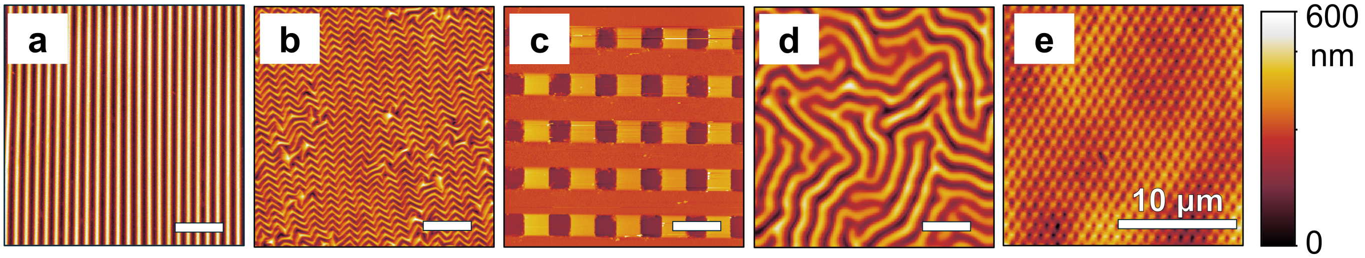


**Figure S5** AFM images of varied pattern mode: a. NOA mode for liner patterns. b. NOA mode chevron patterns. c.AFM calibration mode for square post array. d. PDMS mold for isotropic pattern. e. PDMS mode for checkerboard pattern.

**Morphology of PDMS mode for checkerboard pattern**

All nanolithography processes were conducted using 4-inch (001)-oriented silicon wafers. To minimize standing wave effects in resist sidewalls due to coherent light interference, a bottom anti-reflective coating (BARC) (Wide 30 W, Brewer Science) was spin-coated onto the wafer surface. The BARC deposition involved spinning at 3000 rpm for 30 s, followed by a two-stage bake: 80 °C for 60 s and then 200 °C for 180 s, yielding a uniform 200 nm film. Subsequently, a positive photoresist (PFI) was applied using identical spin-coating parameters. The resist was diluted with EC11 (1:1 ratio) to achieve a target thickness of 280 nm. A post-application soft bake at 90 °C for 90 s ensured proper resist conditioning prior to exposure. The hotplates used exhibited excellent temperature uniformity, as shown in Fig. S6 a and b, guaranteeing consistent and high-quality thermal processing.

The photoresist exposure was carried out using a displacement Talbot lithography (DTL) system (PhableR 100M, EULITHA AG), as illustrated in Fig. S6 c and d. A hexagonal amplitude mask with a 1.5 $\mu$m pitch was employed, along with a laser power density of 1 mW/cm$^2$ and an exposure time of 180 s. To ensure uniform patterning, Gaussian integration was applied along the illumination axis (Z-direction) over twenty Talbot lengths, maintaining a maximum mask-to-wafer gap of 140 $\mu$m. This integration process consisted of two cycles, each lasting roughly 30 s. After exposure, the silicon wafers were subjected to a post-exposure bake (PEB) at 90 °C for 90 s. Subsequently, the samples were allowed to rehydrate for 10 min under ambient conditions. Finally, development was performed using an MF CD-26 solution for 60 s to remove the exposed resist regions.

The surface of the 4-inch mask template was treated with an anti-adhesion layer, as shown in Fig. S6 e

and f. A polydimethylsiloxane (PDMS) prepolymer mixture was cast onto the master and laminated with a polyethylene terephthalate (PET) film to enhance mechanical stability before thermal curing. The resulting flexible PDMS-PET negative replica was then employed in the EVG 620 Semi-automated Nanoimprint Lithography (NIL) system, equipped with Smart NIL tooling for high-precision alignment. During imprinting, the mold was pressed onto a substrate coated with a UV-curable resist under controlled pressure, followed by ultraviolet exposure ($\lambda = 365$ nm) to crosslink the resist. Subsequent demolding yielded PET-PDMS mold with a high-fidelity nanostructured pattern.

Photographs of the fabricated PDMS and NOA molds are presented in Fig. S7. These molds were engineered with high precision to achieve well-defined circular shapes, demonstrating excellent replication fidelity from the master templates. Under illumination with white light-emitting diodes (LEDs), the patterned surfaces exhibited vivid, angle-dependent structural coloration resulting from optical diffraction effects. This coloration confirms the presence of periodic surface features at the sub-micron to micron scale, further validating the quality of the mold fabrication. The observed iridescence also suggests that the patterns have a high degree of uniformity and surface regularity, which are essential characteristics for subsequent nanoimprinting applications.

**Structural design by wrinkling process**

Using Equation 1 from the main text, the predicted periodicity ($d$) curves as a function of the plasma-induced stiff skin thickness ($h$) are presented in Fig. S8. These curves correspond to selected $h$ values of 8, 20, 45, and 63 nm, which were experimentally achieved using the Diener Plasma (Femto) system. The parameter $h$—which plays a critical role in determining the wrinkle wavelength—was precisely controlled by varying key plasma processing parameters, including the plasma power, exposure time, and $O_2$ gas pressure. By systematically tuning these parameters, $h$ was adjusted to desired values, enabling the fabrication of surface wrinkles with tailored periodicities.

To generate target wrinkle periodicities of 0.7, 1, 2, 3, 5, and 7 $\mu$m, specific combinations of mechanical strain, plasma power, exposure time, and oxygen pressure were employed. These experimental conditions are summarized in Table S2. The predictive accuracy of Equation 1 was validated by the close agreement between the calculated and experimentally measured wrinkle periodicities.

In addition, for sub-micron wrinkling, a similar approach was applied using a different plasma system—the Harrick Plasma PDC-002—which is suitable for low-power oxygen plasma treatment. By adjusting processing conditions accordingly, wrinkle periodicities of 0.2 and 0.4 $\mu$m were successfully achieved. These results confirm that the theoretical model is robust across different plasma systems and can be reliably used to guide the design of wrinkled surface morphologies over a broad range of length scales.

Atomic force microscopy (AFM) images of the resulting surface wrinkles on PDMS mode with periodicities of 0.2, 5, and 7 $\mu$m are presented in Fig. S9. The successful fabrication of such diverse periodicities highlights the excellent tunability of the wrinkling process, which can be precisely controlled through the adjustment of

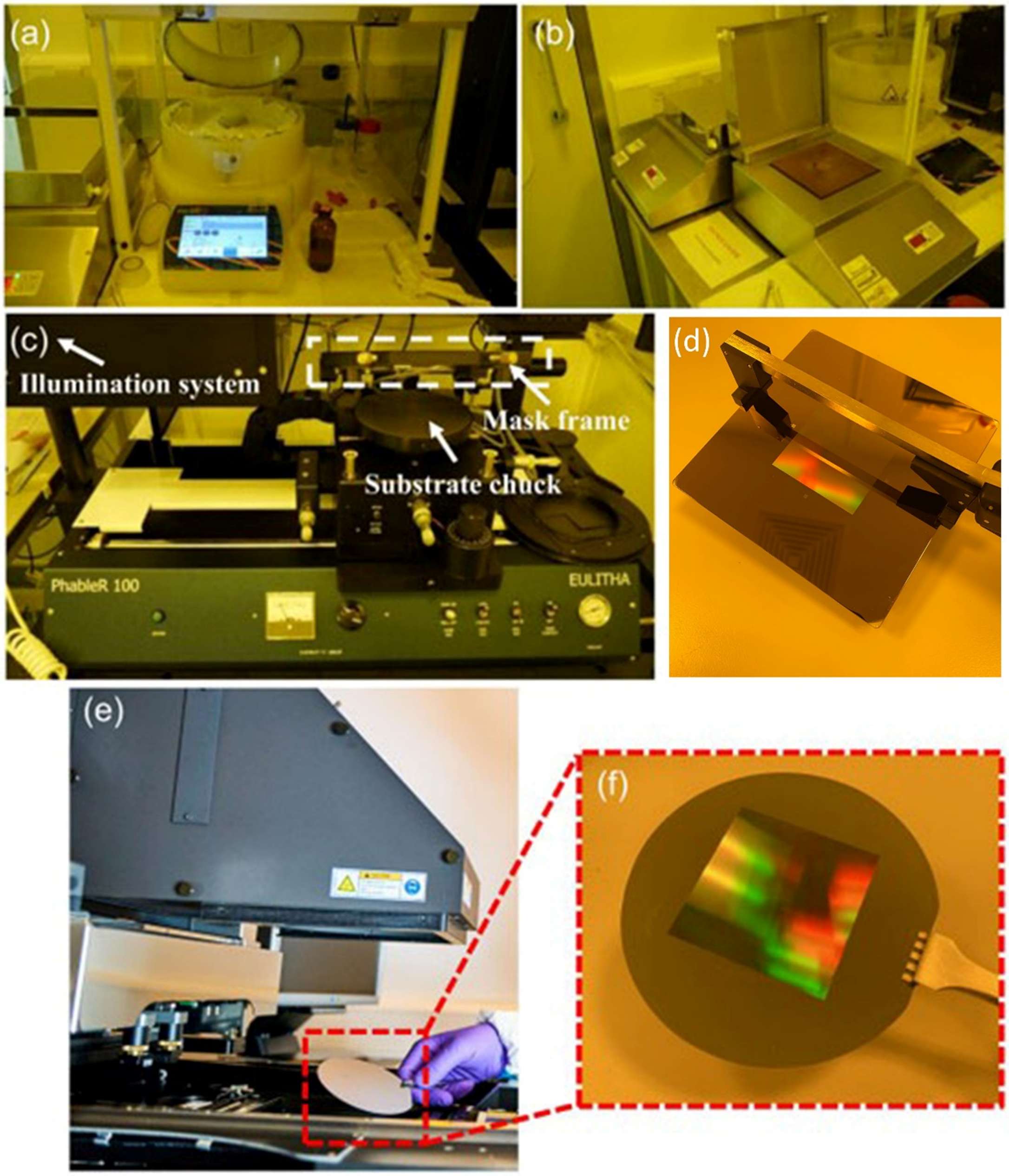


**Figure S6** PET-PDMS mold manufacturing process: PET-PDMS mold manufacturing process: (a) POLOS 150i-NPP single substrate spin processor. (b) Two POLOS 200S table top hotplates. (c) PhableR 100M nanolithography system (d) 1.5 $\mu$m pitch hexagonal amplitude master. (e) EVG 620 Semi-automated Nanoimprint Lithography (NIL) system (f) 1.5 $\mu$m pitch 4-inch hexagonal mask template.

plasma parameters, mechanical strain, and material properties. At the sub-micron scale, the 0.2 $\mu$m periodicity reveals well-aligned and densely packed wrinkle structures, demonstrating the resolution limits achievable with optimized processing conditions. In contrast, the larger-scale patterns with periodicities of 5 and 7 $\mu$m exhibit broader, more relaxed surface undulations, indicating the scalability of the process. Together, these results confirm that the wrinkling method is a versatile and powerful strategy for generating well-ordered, periodic

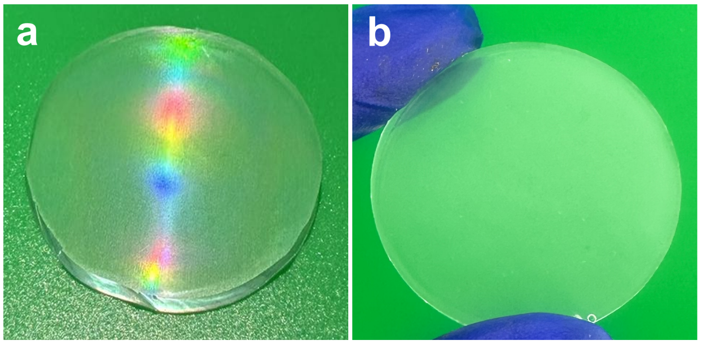


**Figure S7** Photos of PDMS (a) and NOA (b) mold

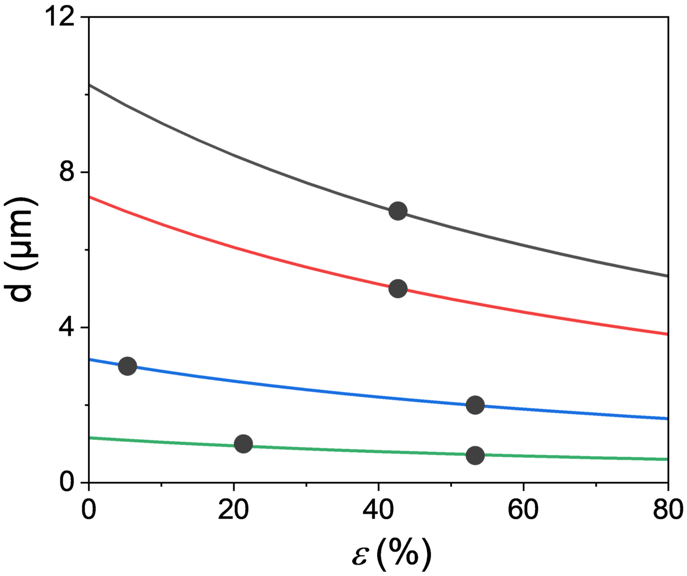


**Figure S8** Theoretical models (colored lines) showing strain (%) as a function of periodicity (d), overlaid with experimental data (black circles) derived from model predictions.

surface topographies for potential applications in optics, surface wetting, adhesion control, and templating technologies.

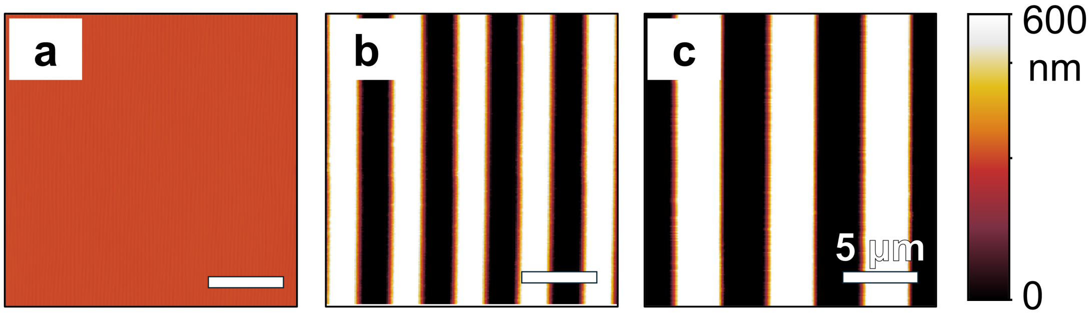


**Figure S9** AFM images of liner NOA mold with periodicity of 0.2, 5 and 7 $\mu$m.

The dimensional characteristics of the fabricated NOA molds, including feature amplitude and periodicity

**Table S2** Processing parameters of PDMS wrinkling method

| Sample | Periodicity ($\mu$m) | Plasma machine | Power (W) | $O_2$ | Plasma time (min) | Strain (%) |
|---|---|---|---|---|---|---|
| W-0.2 | 0.2 | Harrick Plasma | 10.5 mTorr | 490 | 0.75 | 20 |
| W-0.4 | 0.4 | Harrick Plasma | 10.5 mTorr | 380 | 1.5 | 20 |
| W-0.7 | 0.7 | Diener Plasma | 20 | 0.22 mbar | 1 | 50 |
| W-1 | 1 | Diener Plasma | 20 | 0.22 mbar | 1 | 20 |
| W-2 | 2 | Diener Plasma | 99 | 0.22 mbar | 5 | 50 |
| W-3 | 3 | Diener Plasma | 99 | 0.22 mbar | 5 | 5 |
| W-5 | 5 | Diener Plasma | 99 | 0.22 mbar | 10 | 40 |
| W-7 | 7 | Diener Plasma | 99 | 0.22 mbar | 15 | 40 |

are comprehensively summarized in Table S3. These parameters were measured using high-resolution profilometry and atomic force microscopy (AFM) to ensure accuracy and reproducibility. The data provide a quantitative assessment of the mold geometry, confirming that the NOA molds reliably replicate the master templates with minimal distortion or dimensional loss. This level of precision is critical for applications requiring nanoscale pattern transfer, such as nanoimprint lithography, optical component fabrication, and surface functionalization.[13,14]

**Table S3** Geometric information of different NOA molds

| Sample | Periodicity ($\mu$m) | STDEV | Amplitude (nm) | STDEV |
|---|---|---|---|---|
| W-0.2 | 0.2 | 0.02 | 10 | 0.5 |
| W-0.4 | 0.4 | 0.03 | 48 | 2.2 |
| W-0.7 | 0.7 | 0.1 | 107 | 12 |
| W-1 | 1 | 0.1 | 176 | 20 |
| W-2 | 2 | 0.1 | 566 | 25 |
| W-3 | 3 | 0.1 | 624 | 26 |
| W-5 | 5 | 0.1 | 1391 | 32 |
| W-7 | 7 | 0.1 | 2247 | 51 |

The line profile extracted from the AFM image of the nanopatterned CNC film with a periodicity of 7 $\mu$m is presented in Fig. S10. This profile offers a quantitative depiction of the surface morphology, clearly revealing a well-defined sinusoidal shape. The regularity and consistency of the waveform confirm the high fidelity of the mold features and indicate that the surface geometry is well-suited for applying theoretical prediction models based on sinusoidal wrinkle formation.

The smooth, continuous waveform of the profile also indicates minimal surface roughness and edge distortion, both of which are essential for accurate nanoimprinting applications.[15] Such periodic sinusoidal structures are particularly valuable in optical and photonic applications where precise control of surface geometry influences light scattering, diffraction, and interference effects.[13] The successful reproduction of these features further demonstrates the reliability of the fabrication process and the suitability of NOA as a mold material for

high-resolution pattern replication.

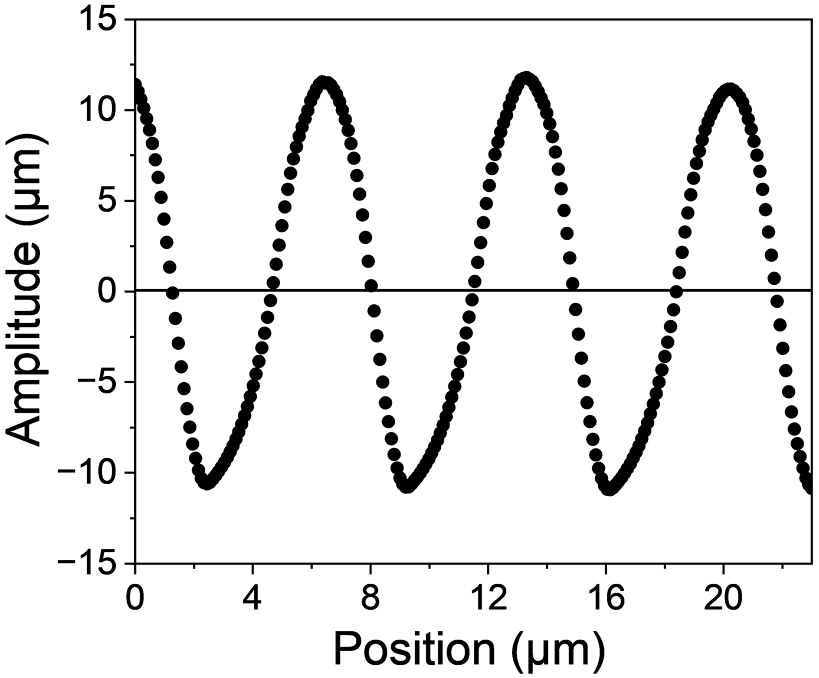


**Figure S10** Sinusoidal line profile from AFM images of nanopatterned CNC films.

## 4 Wet-NIL process modification

### Wet-NIL on CNF and BNC films

The wet nanoimprint lithography (wet-NIL) process was applied to cellulose nanofiber (CNF) and bacterial nanocellulose (BNC) suspensions, each with a concentration of 1 wt%. A mold possessing a well-defined sinusoidal surface pattern, characterized by a periodicity of 2 $\mu$m and an amplitude of 566 nm, was used for imprinting. The process was conducted under elevated temperature and pressure conditions—specifically, at 180 °C and 10 kPa—to promote pattern transfer from the mold to the nanocellulose films.

Despite the use of optimized imprinting parameters, including sufficient thermal energy and pressure to enable viscoelastic deformation of the suspension, neither optical microscopy nor atomic force microscopy (AFM) revealed the presence of well-defined or regular surface patterns on the resulting CNF and BNC films (Fig. S11). While the films retained their macroscopic integrity without cracking or delamination, the absence of distinguishable imprinted structures suggests limitations in pattern transfer efficiency for these specific nanocellulose types.

A likely explanation for this behavior is the intrinsic microstructural characteristics of CNFs and BNC. Both materials consist of long, entangled fibrils with micron-scale lengths, which may hinder their ability to conform to and retain the fine-scale features of the mold. The high aspect ratio and network-forming nature of these fibrils can result in elastic recovery or internal structural resistance during demolding, ultimately prevent-

ing faithful pattern replication. These findings indicate that while wet-NIL is effective for imprinting certain nanocellulose materials, its applicability may be limited by the morphological properties of the target suspension.

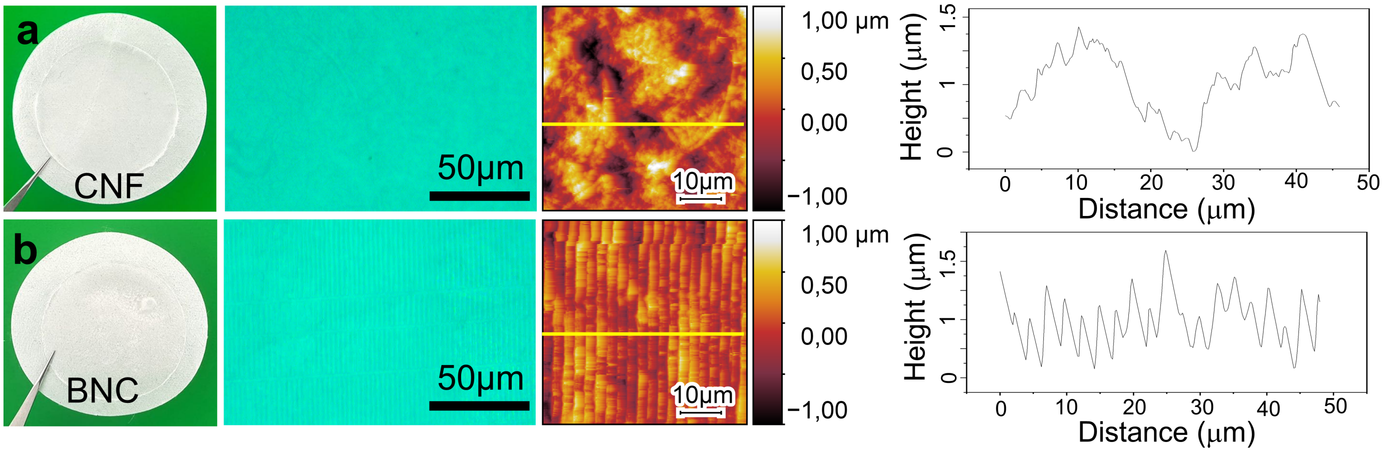


**Figure S11** Photo, microscopy image, AFM image and relevant line profile of nanopatterned CNF (a) and BNC film (b).

**Concentration influence on CNC film nanopatterning**

Concentration plays a critical role in determining both the structural integrity of the CNC films and the fidelity of the replicated surface patterns. As illustrated in Fig. S12, the physical appearance of the films changes significantly across the examined concentration range. At lower concentrations (4–8 wt%), the films appear relatively smooth and flat, with no visible signs of mechanical failure. However, as the concentration increases to 10–12 wt%, noticeable cracking and brittleness begin to emerge, indicating a reduction in film flexibility and mechanical stability at higher solid contents.

To assess the pattern replication performance across different concentrations, atomic force microscopy (AFM) images and corresponding amplitude measurements were obtained, as shown in Fig. S13. At 4 wt%, the surface features exhibit low amplitude and partial pattern transfer, suggesting insufficient material cohesion and mechanical strength for complete mold conformation. Progressive improvements are observed as the concentration increases to 6 wt% and further to 8 wt%, with 8 wt% showing optimal pattern fidelity. At this concentration, the CNC films display highly uniform nanoscale surface structures, with an average amplitude of approximately 521 nm and a consistent periodicity of 2 $\mu$m, indicating successful and reproducible pattern transfer.

In contrast, while nanostructures are still detectable at 10 and 12 wt%, the overall film quality deteriorates due to increased cracking and internal stress, which likely disrupt the pattern uniformity and usability of the films in practical applications. These observations suggest that 8 wt% represents the optimal CNC suspension concentration for wet-NIL processing, offering the best compromise between mechanical stability and high-resolution pattern replication capability in the resulting nanopatterned CNC films.

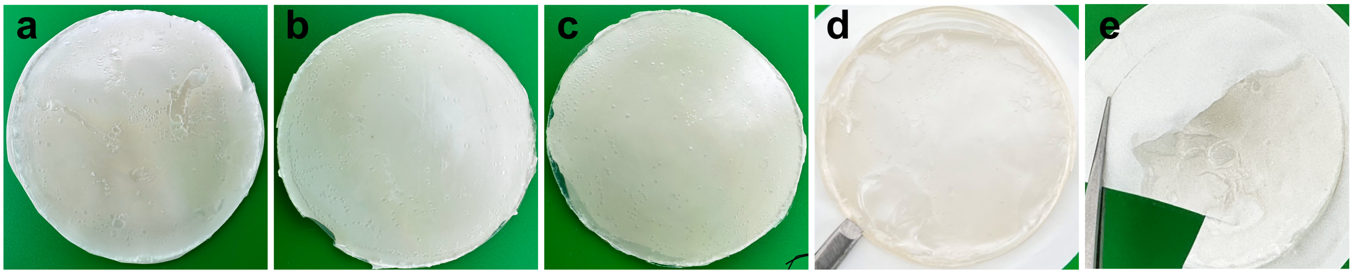


**Figure S12** Photographs of nanopatterned CNC films prepared with varying CNC concentrations: (a) 4 wt%, (b) 6 wt%, (c) 8 wt%, (d) 10 wt%, and (e) 12 wt%.

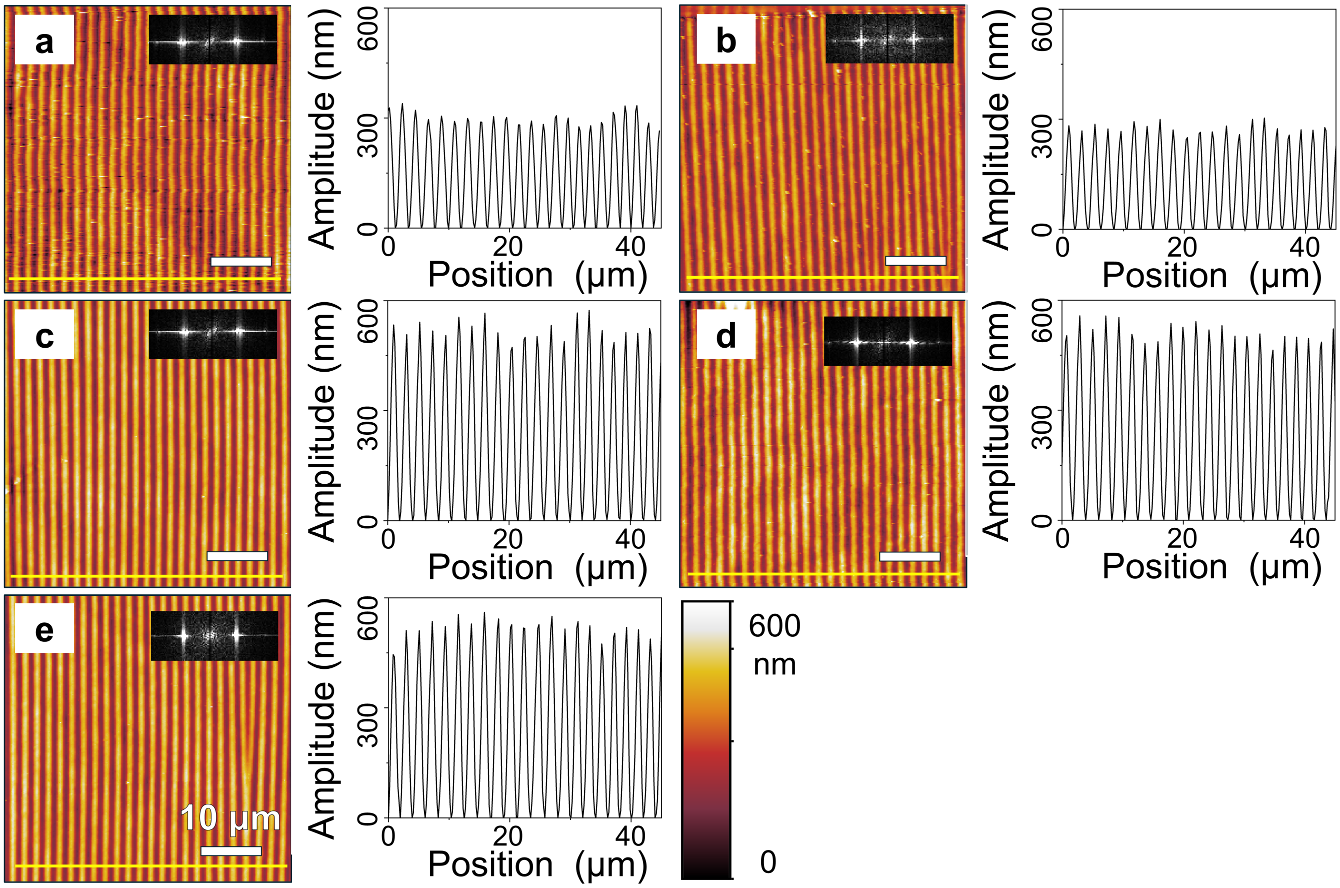


**Figure S13** AFM images and corresponding line profiles of nanopatterned CNC films fabricated at varying CNC concentrations: (a) 4 wt%, (b) 6 wt%, (c) 8 wt%, (d) 10 wt%, and (e) 12 wt%, under 180 °C and 10 kPa in 10 min using a NOA mold with a periodicity of 2 $\mu$m and an amplitude of 566 nm.

## Temperature influence on CNC film nanopatterning

AFM and optical microscopy images acquired at various processing temperatures are presented in Fig. S14 and S15, respectively. These images collectively demonstrate that consistent and well-defined surface patterns were successfully replicated across the full range of investigated thermal conditions. The nanoscale topographies observed in the AFM images confirm that the wet-NIL process is highly robust and capable of producing

uniform structures irrespective of moderate variations in temperature.

To further evaluate the effect of temperature on pattern fidelity, line profiles were extracted from the AFM data. These profiles reveal only slight differences in feature height and periodicity, with all samples maintaining the intended sinusoidal morphology and a consistent periodicity of approximately 2 $\mu$m. The amplitude deviations observed among samples processed at different temperatures were minimal and within acceptable experimental error, suggesting that the thermal processing window for effective pattern transfer is relatively broad.

Despite the process's thermal tolerance, there are practical advantages to operating at the lower end of the temperature spectrum. Specifically, utilizing lower processing temperatures significantly reduces energy consumption, which is particularly beneficial for large-scale or continuous production. Moreover, lower thermal exposure minimizes the risk of thermal degradation of sensitive materials and enhances the overall sustainability of the patterning process. Therefore, while high pattern fidelity can be achieved across a range of temperatures, lower processing temperatures are preferred from both an environmental and economic standpoint.

**Time influence on CNC film nanopatterning**

The influence of processing time on the fidelity of pattern replication was systematically evaluated using atomic force microscopy (AFM). Representative AFM images and their corresponding line profiles, as shown in Fig. S16, reveal negligible differences in the surface topographies obtained at varying imprint durations. Across all tested time intervals, the periodic features remained consistent in both amplitude and wavelength, indicating that the pattern transfer process reaches equilibrium relatively quickly and is not significantly enhanced by prolonged exposure.

This minimal sensitivity to processing time underscores the efficiency and reliability of the wet-NIL process, suggesting that high-quality pattern replication can be achieved without the need for extended imprint durations. From an engineering and manufacturing perspective, this is a particularly valuable finding. Shorter processing times translate directly into increased throughput and reduced operational costs, both of which are critical factors for scaling the process for industrial applications.

Moreover, minimizing the imprint duration can also reduce the thermal and mechanical load on the materials involved, thereby preserving their structural and functional integrity. In summary, while processing time has minimal impact on pattern fidelity, shorter durations are preferred for optimizing process efficiency, cost-effectiveness, and material sustainability in practical implementations of the wet-NIL technique.

**Pressure influence on CNC film nanopatterning**

The applied pressure during the wet-NIL process plays a crucial role in determining the quality of pattern transfer and overall fidelity of the resulting nanostructures. As shown in Fig. S17, AFM images and corresponding line profiles illustrate a clear dependence of pattern replication on the magnitude of applied pressure.

At pressures below 10 kPa, the force exerted by the mold was insufficient to fully imprint the desired

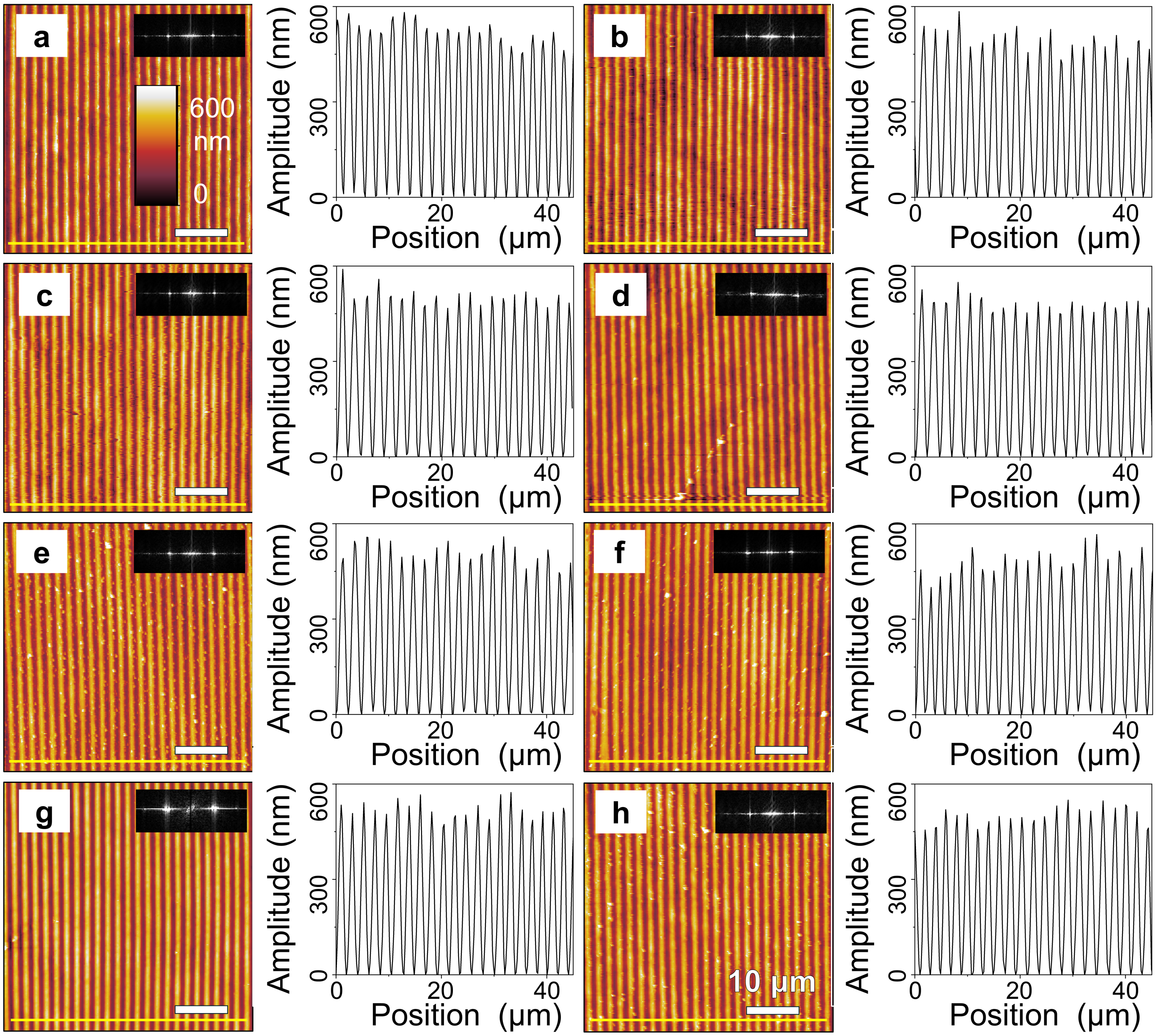


**Figure S14** AFM images and corresponding line profiles of nanopatterned CNC films fabricated at varying process temperatures: (a-h): 20 °C, 40 °C, 60 °C, 90 °C, 120 °C, 150 °C, 180 °C, 220 °C, under 10 kPa with 8 wt% CNC concentration in 10 min, using a NOA mold with a periodicity of 2 $\mu$m and an amplitude of 566 nm.

nanoscale features into the CNC suspension. During the drying phase, the inherent shrinkage and capillary-driven deformation of the CNC network further compromised the fidelity of the imprinted patterns. As a result, the observed surface topography exhibited incomplete feature formation, lower amplitude, and disrupted periodicity. These deviations indicate that the mold was unable to overcome the mechanical resistance of the CNC matrix or maintain uniform contact throughout the drying process under low-pressure conditions.

In contrast, when the applied pressure reached or exceeded 10 kPa, significant improvements in pattern fidelity were consistently observed. Under these conditions, the mold was able to maintain intimate contact with

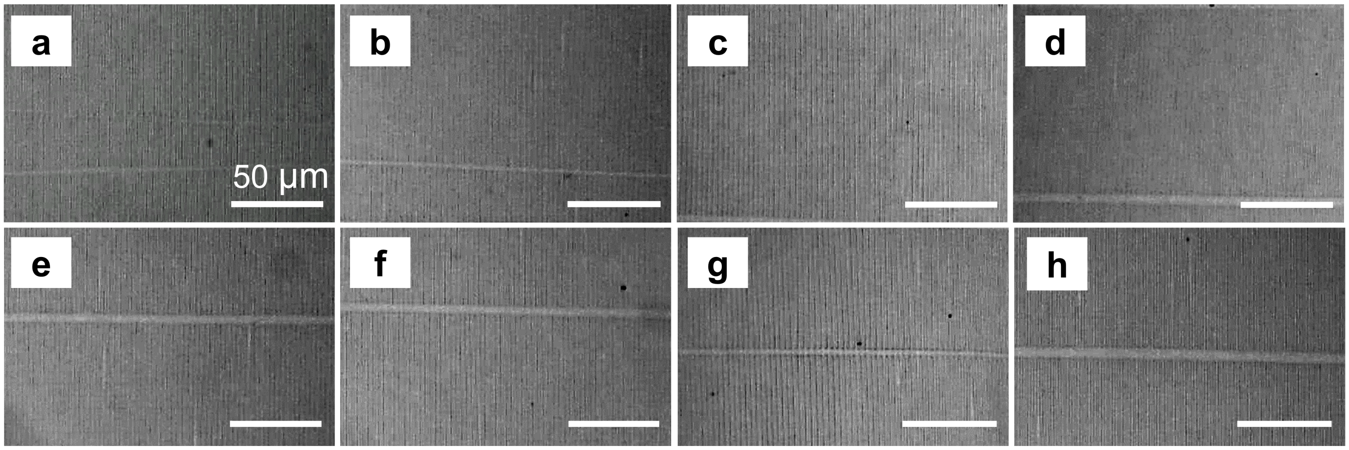


**Figure S15** Microscope images of nanopatterned CNC films fabricated at varying process temperatures: (a-h): 20 °C, 40 °C, 60 °C, 90 °C, 120 °C, 150 °C, 180 °C, 220 °C, under 10 kPa with 8 wt% CNC concentration in 10 min, using a NOA mold with a periodicity of 2 $\mu$m and an amplitude of 566 nm.

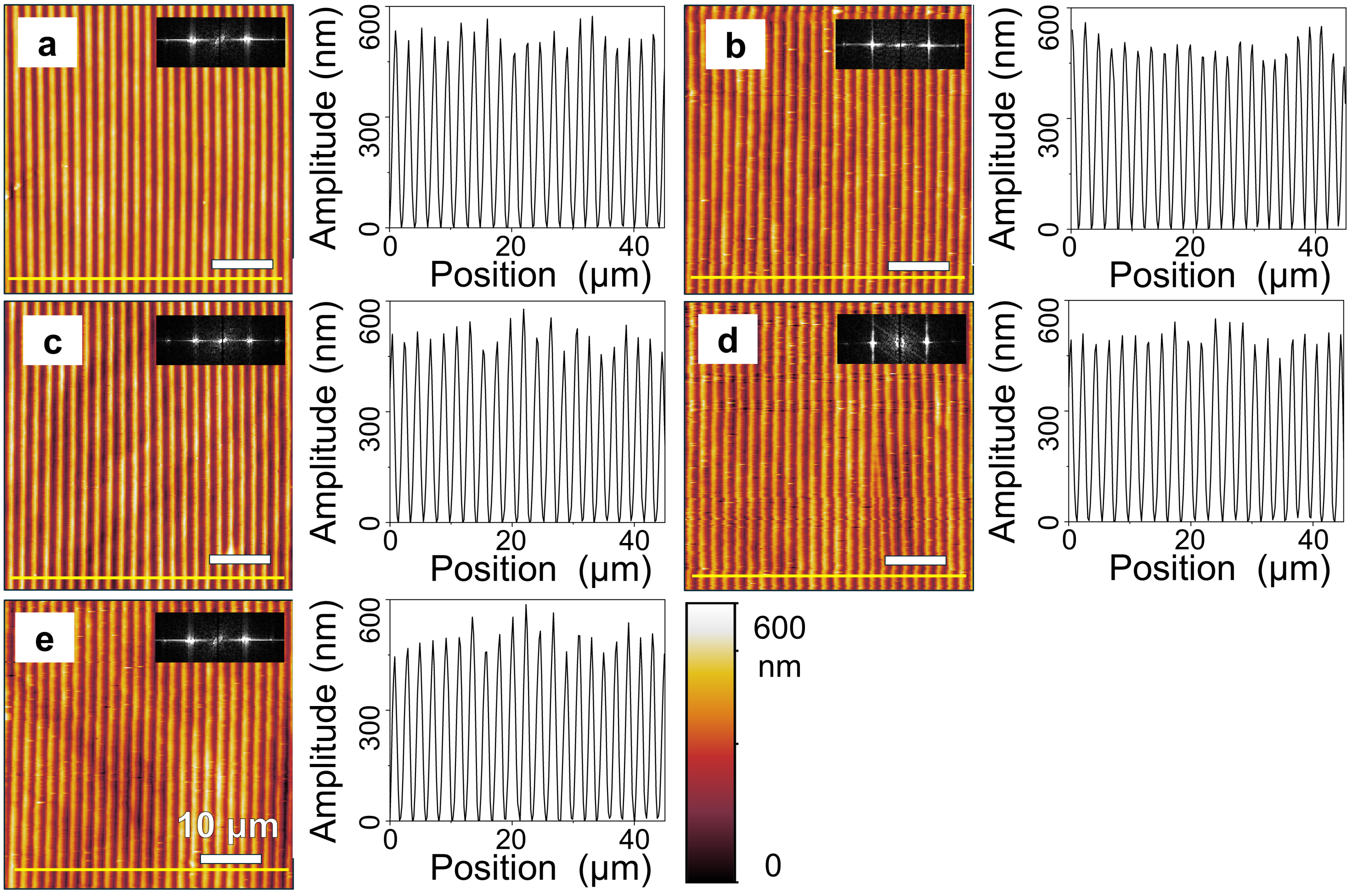


**Figure S16** AFM images and corresponding line profiles of nanopatterned CNC films fabricated at varying process times: (a-e): 10 min, 40 min, 60 min, 80 min, 120 min under 180 °C and 10 kPa with 8 wt% CNC concentration, using a NOA mold with a periodicity of 2 $\mu$m and an amplitude of 566 nm.

the CNC surface throughout the imprinting and drying stages. This ensured that the nanoscale features were faithfully transferred and preserved, as evidenced by the well-defined sinusoidal profiles, uniform amplitudes, and consistent periodicities captured in the AFM analyses.

These findings highlight the importance of applying adequate pressure to counterbalance the mechanical relaxation and shrinkage behavior of CNC films during the wet-NIL process. Consequently, a threshold pressure of at least 10 kPa is recommended to achieve reliable and high-fidelity nanopattern replication.

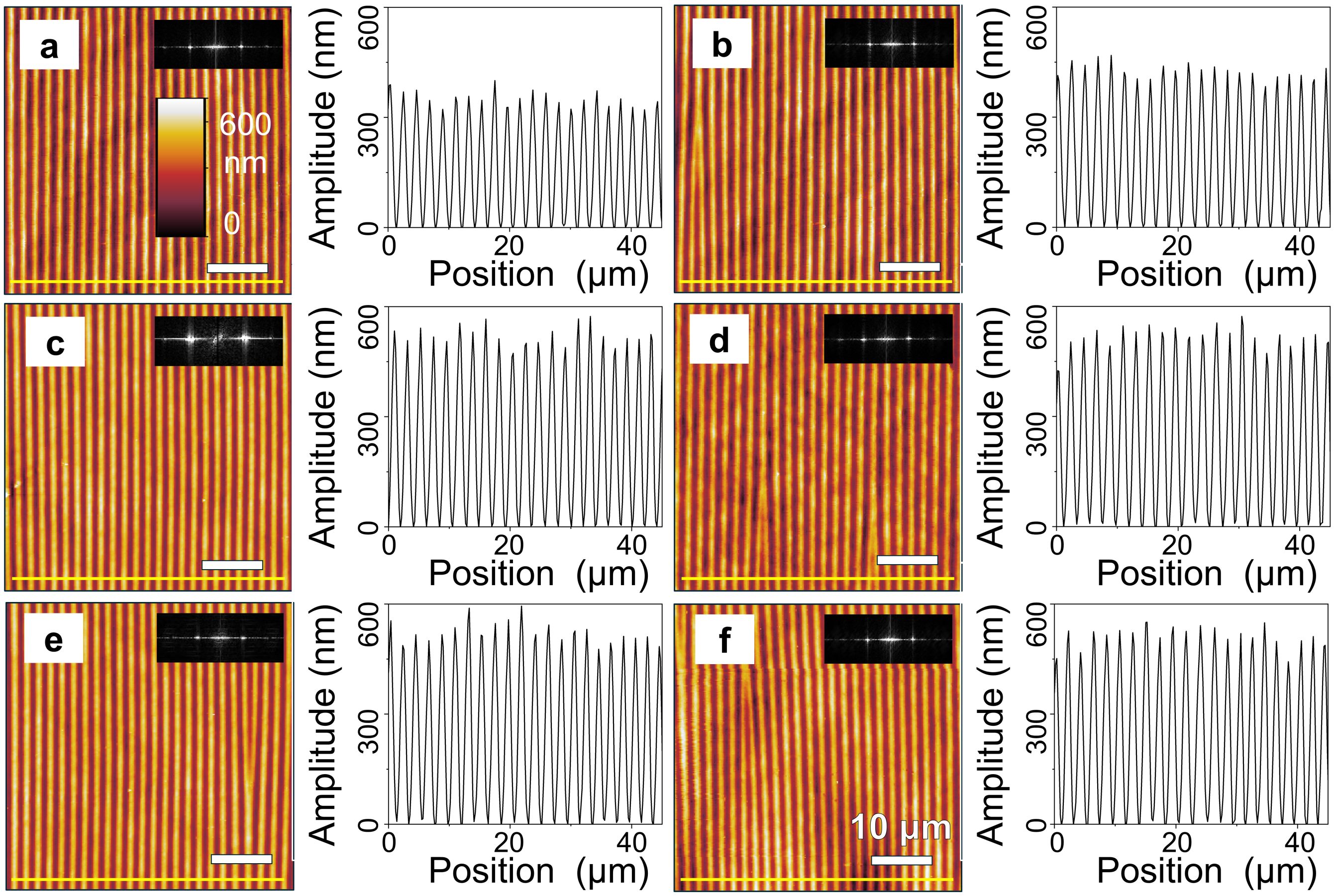


**Figure S17** AFM images and corresponding line profiles of nanopatterned CNC films fabricated at varying pressures: (a-f): 0 kPa, 3 kPa, 10 kPa, 40 kPa, 70 kPa, 100 kPa,under 180 °C with 8 wt% CNC concentration in 10 min, using a NOA mold with a periodicity of 2 $\mu$m and an amplitude of 566 nm.

## 5 Morphology of patterned CNC film with humidity

Figure S18 shows atomic force microscopy (AFM) images comparing the surface morphology of nanopatterned CNC films before and after humidity cycling exposure. The left panel displays the pristine wrinkled surface with well-defined, uniform parallel ridges exhibiting a regular periodicity of approximately 2 $\mu$m. The right panel shows the same sample after humidity cycles, where the wrinkled pattern remains intact with similar periodicity and orientation, demonstrating the structural stability and resilience of the nanopatterned CNC

films under environmental humidity variations. The preserved morphology after humidity cycling suggests good dimensional stability of these nanostructured surfaces.

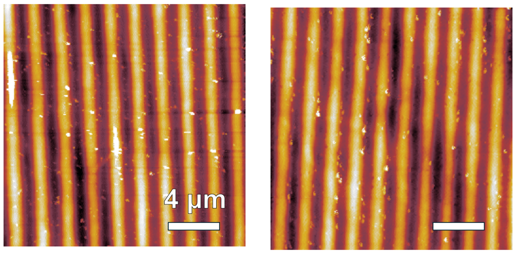


**Figure S18** Morphology of nanopatterned CNC films before and after the humidity cycles.

## 6 Stability of patterned CNC film with water droplet with volumn of 2 $\mu$l

**Method.** To assess the reversibility and stability of the structural color response under water exposure, a 2 $\mu$l water droplet was placed on the nanopatterned CNC film surface and subsequently removed through evaporation (relative humidity around 50%). This wetting-drying cycle was repeated 10 times to evaluate the mechanical and optical stability of the wrinkled surface by SLS.

Figure S19 shows the water droplet cycling tests: (a) shows the normalized first-order diffracted light intensity ($I_1/I_{max}$) response over multiple cycles spanning approximately 15,000 seconds. Each cycle exhibits a characteristic intensity drop upon water addition (to approximately 0% of maximum intensity) followed by recovery to 100% upon evaporation, demonstrating excellent reversibility across at least 10 complete cycles, all showing consistent cyclic behavior. (b) presents real data from a representative first cycle, displaying the temporal evolution of normalized intensity over approximately 1200 seconds, clearly showing the sharp decrease upon water droplet deposition and gradual recovery during evaporation.

Figure S20 shows AFM morphology images of the nanopatterned CNC films before (left) and after (right) water droplet cycles. Both panels reveal well-preserved parallel wrinkle patterns with periodicities of approximately 2 $\mu$m (as indicated by scale bars). The structural integrity of the surface topography is maintained after multiple wetting-drying cycles, confirming that the nanoscale surface features responsible for structural color generation remain intact and functional despite repeated water exposure (2 $\mu$l).

## 7 Stability of patterned CNC film with water droplet with volumn of 100 $\mu$l

**Method.**

Nanopatterned CNC films were precisely sectioned into 1 $cm^2$ samples to facilitate controlled testing and consistent imaging. The initial surface morphology of each sample was characterized using optical microscopy to establish a baseline for pattern fidelity. These baseline images, which depict the as-dried CNC film, are

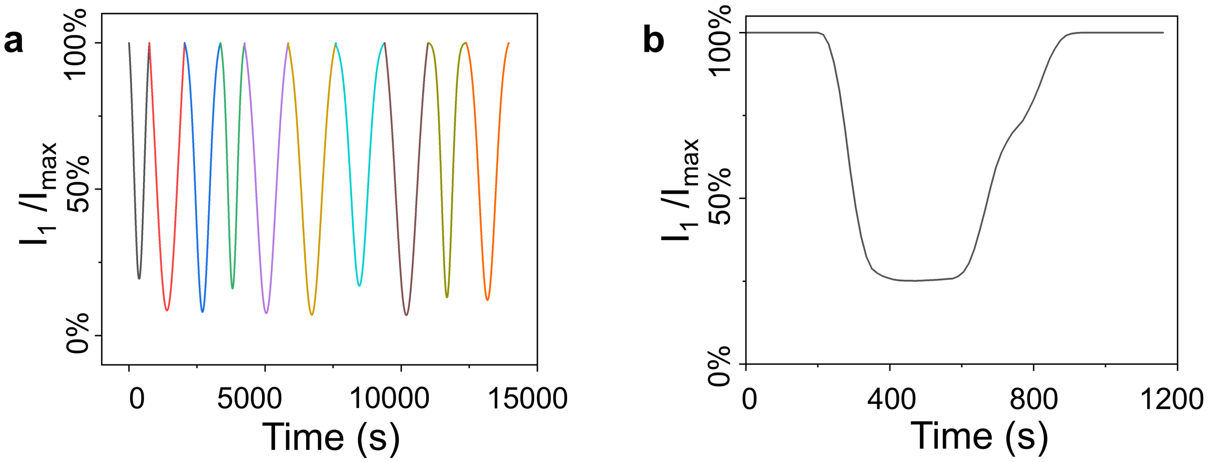


**Figure S19** Water droplet cycling tests: (a) 1st order intensity response (I1/Imax) and (b) real data curve of the first cycle.

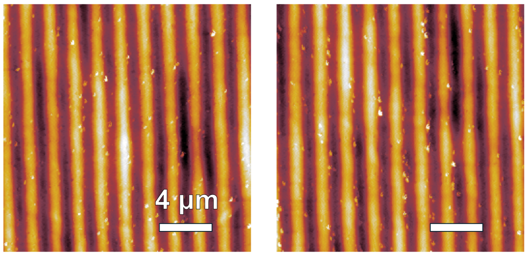


**Figure S20** Morphology of nanopatterned CNC films before and after water droplet cycles.

presented in the first column of Fig. S21 and clearly show well-defined, uniform nanoscale patterns across the imaged areas.

To investigate the stability of the nanopatterns under high-humidity conditions, 100 $\mu$L of deionized (DI) water was carefully deposited onto the surface of each sample to simulate wet exposure. The hydrated films were then transferred into a humidity chamber to allow controlled moisture absorption for varying durations: 15 minutes, 2 hours, 4 hours, and 6 hours. The humidity chamber was established using a saturated sodium sulfate ($Na_2SO_4$) solution, which effectively maintained a consistent relative humidity level of approximately 95 %, ensuring reproducible environmental conditions during the exposure period.

After each designated time point, the same region of each sample was re-examined using optical microscopy to assess the evolution of the surface pattern under wet conditions. The images captured during these post-exposure observations are displayed in the second column of Fig. S21. Any changes in surface morphology, such as blurring of features, pattern deformation, or loss of uniformity, were carefully documented to evaluate the extent of structural integrity degradation due to moisture absorption.

To further analyze the reversibility and resilience of the patterns, the wetted samples were subjected to a

drying step at 75 °C for 3 minutes. After drying, the exact same areas previously imaged were again examined via optical microscopy. The resulting images, shown in the third column of Fig. S21, provide insights into the ability of the CNC films to recover their original structure post-drying and reveal the degree of permanent deformation, if any. This comprehensive evaluation offers a clear understanding of the environmental durability of nanopatterned CNC surfaces and their potential applicability in water-rich environments.

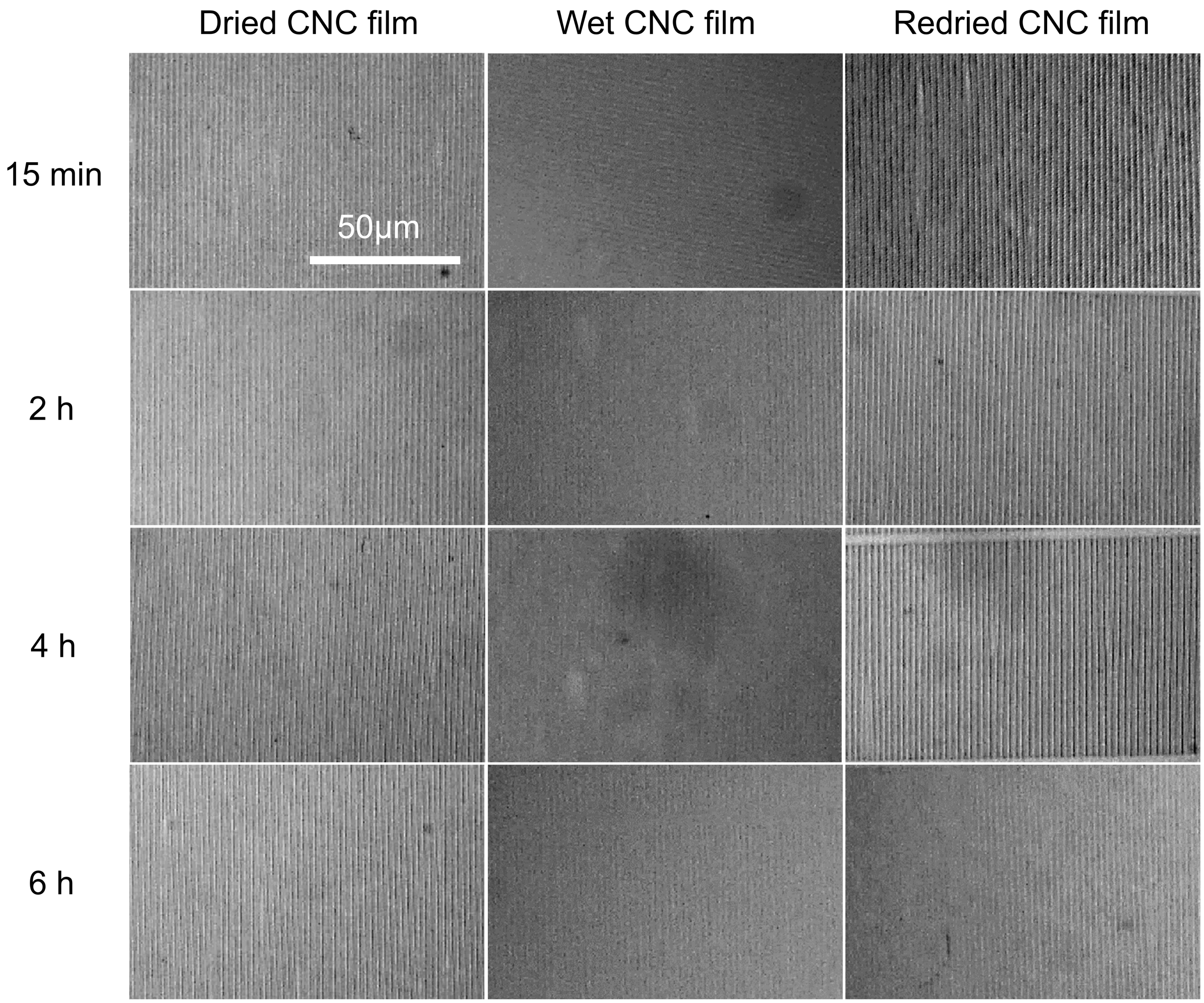


**Figure S21** Morphological evolution of CNC films with linear nanopatterning under different hydration states (dried, wet, redried) observed over time intervals (15 min, 2 h, 4 h, 6 h).

Fig. S21 provides a comprehensive visualization of the effect of water exposure and subsequent drying on the surface morphology of nanopatterned CNC films. Upon the application of 100 $\mu$L of deionized water, the initial surface patterns undergo visible changes, attributed primarily to the swelling behavior of the CNC matrix. This swelling is induced by water absorption into the hydrophilic nanocellulose network, which results in partial deformation of the imprinted structures. As shown in the second column of Fig. S18, although the patterns become less defined due to surface softening and slight structural distortion, the overall periodicity and form

of the features remain detectable under optical microscopy. This indicates that the structural framework of the pattern is not completely lost during hydration.

Following exposure to high humidity for different durations (15 minutes, 2 hours, 4 hours, and 6 hours), and subsequent redrying at 75 °C for 3 minutes, a notable recovery of surface morphology is observed. The third column of Fig. S21 shows that, after redrying, the nanoscale features re-emerge with clarity. Interestingly, for the samples exposed for shorter durations (15 minutes, 2 hours, and 4 hours), the recovered patterns not only regained their shape but in some cases appeared even more sharply defined than in their original state. This phenomenon suggests a possible rearrangement of CNCs during the hydration–dehydration cycle, where the CNC mobility introduced by water uptake may facilitate local alignment of nanocrystals, thereby enhancing pattern definition upon drying.

For the sample exposed for 6 hours, although the pattern is still present, a slight reduction in uniformity and fidelity is noticeable, indicating the onset of irreversible deformation when exposed to prolonged moisture. Overall, this behavior demonstrates that the surface morphology of the CNC films achieves a certain level of resilience and semi-reversibility. The ability of the nanopatterns to withstand short- to medium-term humidity exposure while maintaining recoverability upon drying suggests potential utility in environments subject to intermittent moisture, provided the exposure remains within a tolerable duration and intensity.

**Table S4** Geometric information of different liner nanopatterned CNC films

| Sample | Periodicity ($\mu$m) | STDEV | Amplitude (nm) | STDEV |
|---|---|---|---|---|
| W-0.2 | 0.2 | 0.02 | 9 | 0.5 |
| W-0.4 | 0.4 | 0.03 | 44 | 2.4 |
| W-0.7 | 0.7 | 0.1 | 98 | 14 |
| W-1 | 1 | 0.1 | 162 | 23 |
| W-2 | 2 | 0.1 | 521 | 25 |
| W-3 | 3 | 0.1 | 574 | 29 |
| W-5 | 5 | 0.1 | 1280 | 35 |
| W-7 | 7 | 0.1 | 2067 | 61 |

# 8 Mophology of liner patterned CNC films

Fig. S22 presents AFM images of nanopatterned CNC films featuring three distinct periodicities: 0.2 $\mu$m, 5 $\mu$m, and 7 $\mu$m. These images clearly demonstrate the excellent replication capability of the wet-NIL process across a wide range of length scales, from the sub-micron to multi-micron regimes. For each periodicity, the transferred surface structures exhibit high fidelity to the original mold patterns, as evidenced by the uniformity, consistency, and resolution of the sinusoidal features observed in the topographical scans.

In the 0.2 $\mu$m case, fine nanostructures are precisely resolved, showcasing the wet-NIL technique's ability to duplicate delicate, high-resolution features with minimal distortion. This highlights the effectiveness of the

process in applications requiring fine nanoscale control. The 5 $\mu$m and 7 $\mu$m patterned films similarly display well-defined periodic topographies, confirming that the process is equally effective for larger feature sizes. The replication remains consistent across the surface, indicating uniform mold–substrate contact and efficient pattern transfer during the imprinting stage.

The dimensional characteristics of these patterned films, including measured periodicities and amplitudes are quantitatively summarized in Table S4. These data further confirm the accuracy and repeatability of the patterning process. Collectively, the results illustrate the versatility and robustness of the wet-NIL technique in producing high-fidelity, nanopatterned CNC films over a broad spectrum of geometries, positioning it as a powerful tool for fabricating functional bio-based nanostructures for optics, sensors, and advanced coatings.

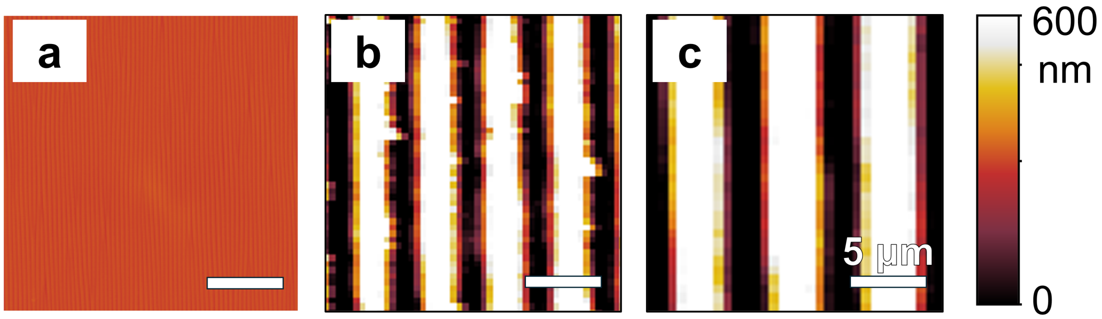


**Figure S22** AFM images of nanopatterned CNC films with pattern periodicity of 0.2, 5 and 7 $\mu$m.

Scanning electron microscopy (SEM) images were obtained with a Zeiss Auriga microscope. All films were sputtered with gold before measurements. Figure S23 presents SEM images comparing the surface morphology of pure CNC film (top left) and nanopatterned CNC films with systematically varied wrinkle periodicities. The pure CNC film exhibits a smooth, featureless surface without any periodic structure. In contrast, the nanopatterned samples display well-defined, parallel wrinkle patterns with periodicities of 0.2, 0.4, 0.7, 1, 2, 3, 5, and 7 $\mu$m (shown left to right across the top and bottom rows). As the periodicity increases, the wrinkles become more widely spaced and more prominent. The samples with smaller periodicities (0.2-0.4 $\mu$m) show tightly packed, fine striations, while those with intermediate periodicities (0.7-2 $\mu$m) exhibit clearly resolved parallel ridges ideal for visible light diffraction. The largest periodicities (3-7 $\mu$m) display broader, more pronounced wrinkle features with increased spacing between adjacent peaks. These SEM images demonstrate precise control over the wrinkle periodicity through plasma oxidation parameters, enabling systematic tuning of the structural color across the visible spectrum. The uniformity and regularity of the wrinkle patterns across all periodicities confirm the robust and controllable nature of the fabrication method for creating structural color in CNC-based materials. However, because of the gold sputtering, SEM can not be used to evaluate the pattern's amplitude.

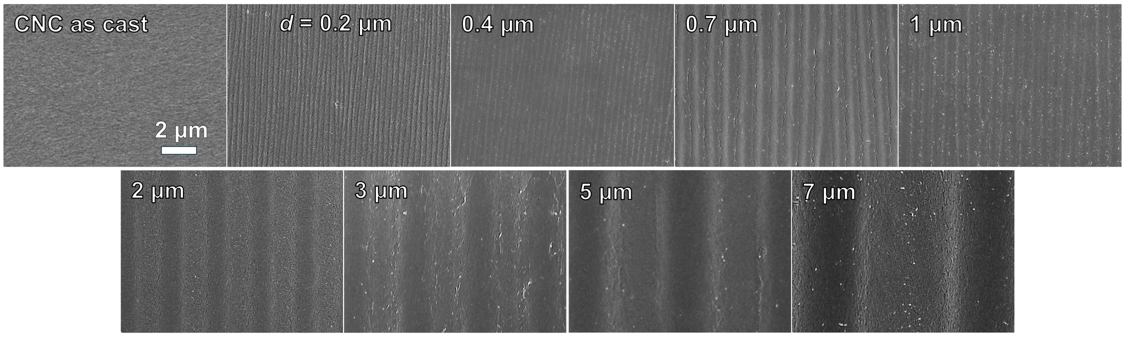


**Figure S23** SEM images of pure CNC film and nanopatterned CNC films with pattern periodicity of 0.2, 0.4, 0.7, 1, 2, 3, 5 and 7 $\mu$m.

# 9 Modeling color intensity

Fig. S24 presents the theoretical diffraction intensity as a function of surface amplitude for different diffraction orders ($n = 0$ to $n = 4$) under incident light of three wavelengths: (a) 380 nm, (b) 532 nm, and (c) 780 nm. For each wavelength, the left column displays the normalized intensity on a linear scale, while the right column presents the same data on a logarithmic scale to highlight differences across a wide dynamic range.

These calculations were performed to model sinusoidal surface gratings using Eq. 3 (main text), assuming normal light incidence and coherent diffraction. The results reveal a periodic modulation of diffraction intensity with increasing amplitude, strongly dependent on both wavelength and diffraction order.

For shorter wavelengths (Fig. S24a, 380 nm), significant diffraction into higher orders occurs at relatively lower amplitudes. As the wavelength increases (Fig. S24b and S24c), the amplitude required to achieve comparable diffraction intensity in higher orders also increases, indicating a shift in optimal grating depth for effective light manipulation. The zero-order (specular reflection) intensity generally decreases with increasing amplitude, while higher-order intensities emerge and then oscillate due to constructive and destructive interference conditions.

The logarithmic plots (right panels) provide enhanced clarity for small-intensity features and show the complex evolution of each diffraction order. These theoretical trends confirm that both the choice of wavelength and the surface amplitude must be carefully tuned to maximize desired diffraction efficiency in CNC-based photonic surfaces. This insight aids in the rational design of nanostructures for optical filtering, structural coloration, and wavelength-selective sensing.

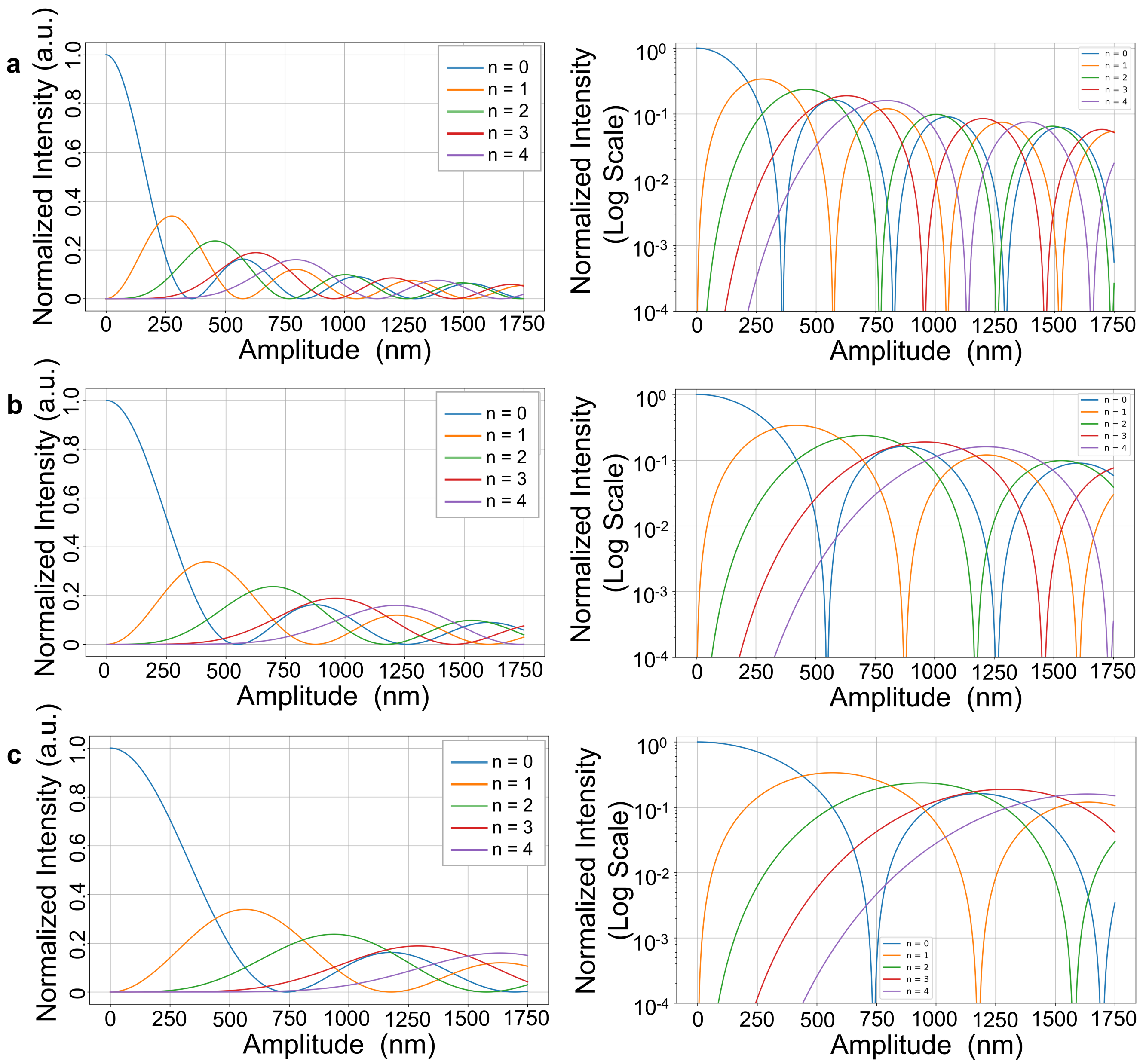


**Figure S24** Theoretical diffraction intensity models as a function of amplitude for varying light wavelengths: (a) 380 nm, (b) 532 nm and (c) 780 nm (log scale on the right side).

# References

[1] M. S. Reid, M. Villalobos and E. D. Cranston, *Langmuir*, 2017, **33**, 1583–1598.

[2] O. M. Vanderfleet and E. D. Cranston, *Nature Reviews Materials*, 2021, **6**, 124–144.

[3] A. Isogai and L. Bergström, *Current Opinion in Green and Sustainable Chemistry*, 2018, **12**, 15–21.

[4] M. Gao, J. Li, Z. Bao, M. Hu, R. Nian, D. Feng, D. An, X. Li, M. Xian and H. Zhang, *Nature Communications*, 2019, **10**, 437.

[5] Y. Habibi, L. A. Lucia and O. J. Rojas, *Chemical Reviews*, 2010, **110**, 3479–3500.

[6] F. Rol, M. N. Belgacem, A. Gandini and J. Bras, *Progress in Polymer Science*, 2019, **88**, 241–264.

[7] T. Abitbol, A. Rivkin, Y. Cao, Y. Nevo, E. Abraham, T. Ben-Shalom, S. Lapidot and O. Shoseyov, *Current Opinion in Biotechnology*, 2016, **39**, 76–88.

[8] Y. Nishiyama, P. Langan and H. Chanzy, *Journal of the American Chemical Society*, 2002, **124**, 9074–9082.

[9] C. G. Vonk, *Journal of Applied Crystallography*, 1973, **6**, 148–152.

[10] W. Bai, J. Holbery and K. Li, *Cellulose*, 2009, **16**, 455–465.

[11] I. Siró and D. Plackett, *Cellulose*, 2010, **17**, 459–494.

[12] A. Dufresne, *Materials Today*, 2013, **16**, 220–227.

[13] D. K. Oh, T. Lee, B. Ko, T. Badloe, J. G. Ok and J. Rho, *Frontiers of Optoelectronics*, 2021, **14**, 229–251.

[14] L. Zheng, U. Zywietz, T. Birr, M. Duderstadt, L. Overmeyer, B. Roth and C. Reinhardt, *Microsystems & Nanoengineering*, 2021, **7**, 1–11.

[15] M. Li, Y. Chen, W. Luo and X. Cheng, *Micromachines*, 2021, **12**, 349.
